# Supplementary material for: Population genetic models of GERP scores suggest pervasive turnover of constrained sites across mammalian evolution
Source: PLoS Genet. 2020 May 29;16(5):e1008827. doi: 10.1371/journal.pgen.1008827 (PMC7286533; doi:10.1371/journal.pgen.1008827)
Supplement: S1 Text — (PDF) [file pgen.1008827.s001.pdf]

S1 Text. Model assumptions and robustness of inference

The GERP score quantifies constraint at individual alignment positions in terms of "rejected substitutions" (RS), defined as the number of substitutions expected under neutrality minus the number of substitutions "observed" at the position [1,2]. Positive scores represent a substitution deficit, which would be expected for sites under selective constraint. To estimate RS, the software estimates a scaling factor, applied uniformly to all branches of the tree, that maximizes the probability of the observed nucleotides in the alignment column. The estimation assumes the HKY85 [3] model of nucleotide evolution, although the original GERP study suggests that alternative realistic models have negligible impacts on the results [1]. Further, a uniform scaling factor assumes that the strength of selection does not change across the phylogenetic tree, whereas changes in population size can lead to variation in the substitution rate of deleterious mutations.

Here, we provide further evidence for the robustness of our inference to specific model assumptions. First, we explore how different assumptions about the transition to transversion (tr/tv) ratio and the GC content affect the GERP score distribution. The transition to transversion ratio for human intergenic regions was estimated to be ~2 [4], but is higher for genes and was consistently estimated to be ~4 among multiple mammalian lineages [5]. The GC content of mammalian genomes varies between 40% and 50% [6]. We simulated alignment data under the HKY85 model and all combinations of tr/tv ratio (2 vs. 4) and GC content (40% vs. 50%). The simulations under these different parameter combinations result in GERP score distributions that are almost identical (S13 Fig). Moreover, we derive similar results when simulating under a GTR model with parameters estimated for mammals [7] (S13 Fig). We thus conclude that our results are robust to assumptions about the nucleotide evolution model.

Our simulation of deleterious substitutions follows the framework developed in Nielsen and Yang [8]. It assumes that there is no interference in the fixation process of multiple mutations at different sites, that there are never more than two alleles segregating at the same nucleotide sites, and that the selection coefficient acting on new mutations at a site is constant in a particular lineage. These assumptions are most likely valid in all organisms that we consider, in particular when considering deleterious mutations [8].

Finally, we explored the effect of changes in the effective population size across the phylogenetic tree on the distribution of GERP scores. Since different mammalian species vary in their effective population size, and the effective population size affects the fixation probability (see eq. 1), large variation in effective population size across the phylogenetic tree may

exaggerate the effect of functional turnover. We estimated the effective population size of each species in the 36 mammalian species tree based on neutral genetic diversity and body weight. First, we collected estimates of synonymous genetic diversity from the literature [9,10]. For species where we did not find an estimate of synonymous diversity, we predicted diversity from its body weight from a linear regression model of log(bodyweight) on log(diversity) (S5 Fig). Bodyweight for all 36 species was extracted from the AnAge database [11]. The effective population size was then calculated for each species by assuming a perfect linear relationship between neutral diversity and effective population size, and assuming a human effective population size of 20,000 and a mouse effective population size of 580,000 [12]. The population size at ancestral states was then estimated using a maximum likelihood approach implemented in the function fastAnc of the R phytools package [13], assuming a Brownian model of the evolution of population size along a phylogenetic tree (S6 Fig). Simulations of GERP scores under this population size change model show very similar results (S7 Fig) to simulations under the constant population size model (Fig 3). The relationship between $N_e s$ and GERP score is not substantially affected by realistic changes in population size across the phylogenetic tree.

References:

1. Cooper GM, Stone EA, Asimenos G, NISC Comparative Sequencing Program, Green ED, Batzoglou S, et al. Distribution and intensity of constraint in mammalian genomic sequence. Genome Res. 2005;15: 901–913.

2. Davydov EV, Goode DL, Sirota M, Cooper GM, Sidow A, Batzoglou S. Identifying a High Fraction of the Human Genome to be under Selective Constraint Using GERP++. PLoS Comput Biol. 2010;6: e1001025.

3. Hasegawa M, Kishino H, Yano T. Dating of the human-ape splitting by a molecular clock of mitochondrial DNA. J Mol Evol. 1985;22: 160–174.

4. Bainbridge MN, Wang M, Wu Y, Newsham I, Muzny DM, Jefferies JL, et al. Targeted enrichment beyond the consensus coding DNA sequence exome reveals exons with higher variant densities. Genome Biol. 2011;12: R68.

5. Rosenberg MS, Subramanian S, Kumar S. Patterns of transitional mutation biases within and among mammalian genomes. Mol Biol Evol. 2003;20: 988–993.

6. Romiguier J, Ranwez V, Douzery EJP, Galtier N. Contrasting GC-content dynamics across 33 mammalian genomes: relationship with life-history traits and chromosome sizes. Genome Res. 2010;20: 1001–1009.

7. Upham NS, Esselstyn JA, Jetz W. Inferring the mammal tree: Species-level sets of phylogenies for questions in ecology, evolution, and conservation. PLoS Biol. 2019;17: e3000494.

8. Nielsen R, Yang Z. Estimating the distribution of selection coefficients from phylogenetic data with applications to mitochondrial and viral DNA. Mol Biol Evol. 2003;20: 1231–1239.

9. Osada N. Genetic diversity in humans and non-human primates and its evolutionary consequences. Genes Genet Syst. 2015;90: 133–145.

10. Leffler EM, Bullaughey K, Matute DR, Meyer WK, Ségurel L, Venkat A, et al. Revisiting an old riddle: what determines genetic diversity levels within species? PLoS Biol. 2012;10: e1001388.

11. Tacutu R, Thornton D, Johnson E, Budovsky A, Barardo D, Craig T, et al. Human Ageing Genomic Resources: new and updated databases. Nucleic Acids Res. 2018;46: D1083–D1090.

12. Halligan DL, Oliver F, Eyre-Walker A, Harr B, Keightley PD. Evidence for Pervasive Adaptive Protein Evolution in Wild Mice. PLoS Genet. 2010;6: e1000825.

13. Revell LJ. phytools: an R package for phylogenetic comparative biology (and other things). Methods in Ecology and Evolution. 2012. pp. 217–223.
