# Supplementary material for: Population genetic models of GERP scores suggest pervasive turnover of constrained sites across mammalian evolution
Source: PLoS Genet. 2020 May 29;16(5):e1008827. doi: 10.1371/journal.pgen.1008827 (PMC7286533; doi:10.1371/journal.pgen.1008827)
Supplement: S2 Text — (PDF) [file pgen.1008827.s002.pdf]

S2 Text. Optimally increasing power when adding species

When selection acts uniformly across the whole phylogenetic tree, then adding new sequence data from more species always increases power for detecting sites under selective constraint in a certain focal species. Assuming that the alignment of the new sequence is possible and substitutions can be reliably detected, this further suggests that adding a species that is highly diverged to the focal species would increase power more than adding a closely related species, since it would lead to a larger increase in the expected number of neutral substitutions per site and thus to a higher probability to detect a lack of substitutions caused by negative selection. However, under a functional turnover model, species that are more closely related to the focal species might be more valuable since they are more likely to share the functional state of the focal species than more distantly related species. To explore this tradeoff between sequence divergence and functional state turnover on the power to detect selection, we simulated neutral and selected substitutions on a conceptual phylogenetic tree (S14 Fig) and evaluate the change in power when adding additional species with varying degree of relatedness to the focal species (S14A-D Fig). First, we confirm that in the case of no turnover, power increases most if the added species has highest divergence to the focal species (S15B Fig). Next, we simulate functional turnover in the same way as we did for the results in Fig 4. Under the turnover model, adding species with an intermediate level of relatedness (0.4 subs/site; e.g. equivalent to adding species with divergence to humans similar to mouse, rat, etc.) leads to a large increase in power per added species (S15A Fig). There is almost no added value when adding highly diverged species and power plateaus at very low levels (S15B Fig; 2 subs/site, e.g. adding species with divergence to humans similar to lamprey or zebrafish). This result is concordant with what we found in Fig 4, where, after an optimum at tree size ~6 subs/site, adding more and more diverged species decreases the power by adding noise to the GERP score distribution. If the added species are too closely related, then power increases steadily but only very slowly (S15C Fig; 0.1 subs/site, e.g., adding primate data such as baboons, macaques, or marmosets). Roughly, the increase in power when adding the mouse sequence is twice the increase in power when adding a primate sequence. Finally, we also note that adding species that are closely related to an already sampled species (e.g. adding mouse data when rat is already included) again does not substantially increase power (S15D Fig).
