# Supplementary figures and images for: Population genetic models of GERP scores suggest pervasive turnover of constrained sites across mammalian evolution
Source: PLoS Genet. 2020 May 29;16(5):e1008827. doi: 10.1371/journal.pgen.1008827 (PMC7286533; doi:10.1371/journal.pgen.1008827)

$-Nes$ 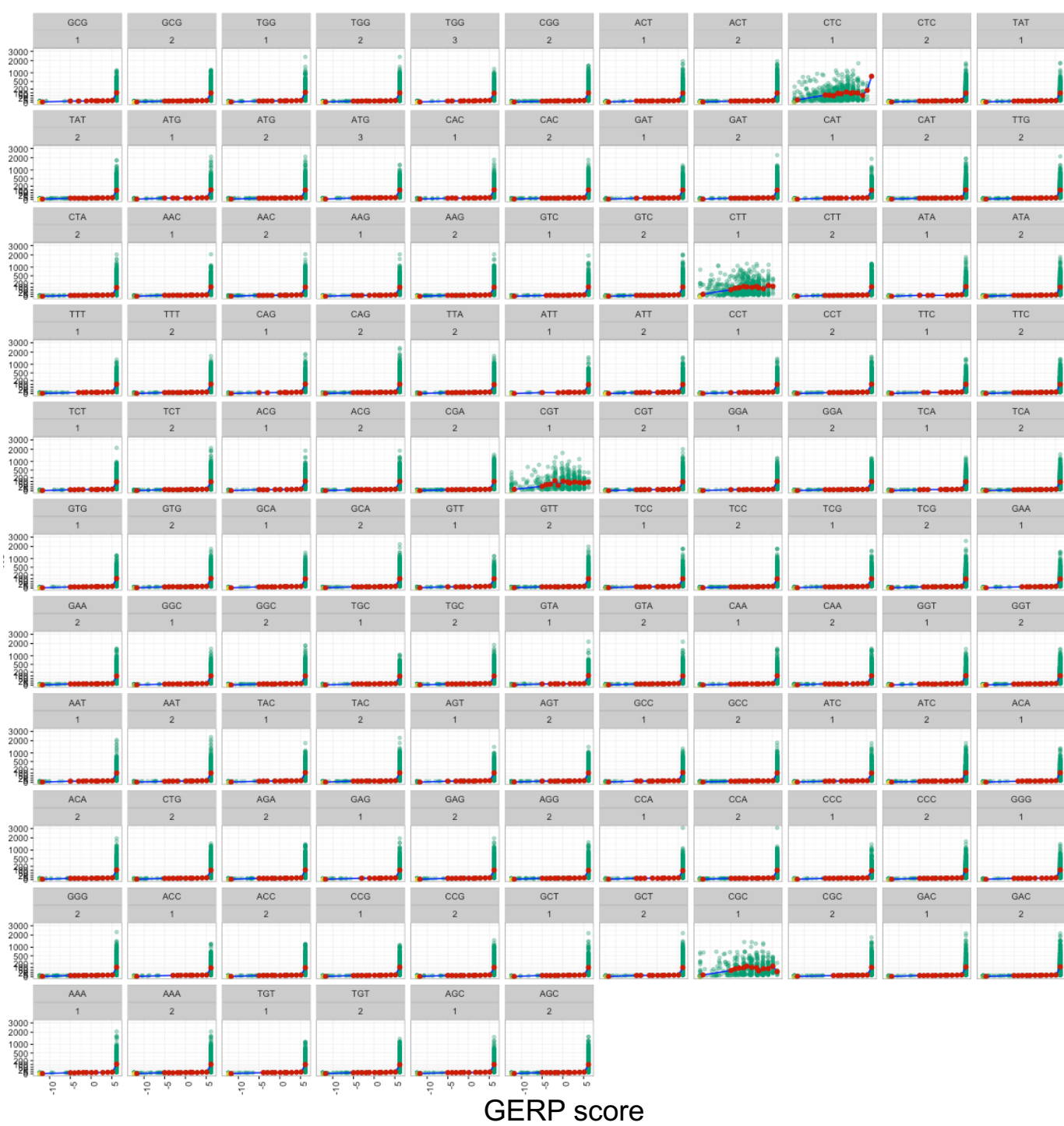

Supplement: S1 Fig — The strength of selection, Nes, is distributed according to a mixture distribution where 82.6% of mutations have gamma-distributed Nes values (shape = 0.343, scale = 334) and 17.4% of mutations are neutral. The green points represent selected mutations, the blue points neutral mutations. The red points are averages of Nes in bins of GERP scores. Note that the first codon position of four codons (CTC, CTT, CGT, CGC) shows a less consistent relationship between GERP score and strength of selection where even strongly selected sites can have GERP scores that are different from the maximum value. We thus classified these four classes of sites as two-fold instead of zero-fold. (PDF) [file pgen.1008827.s003.pdf]

## Coding

## Intergenic

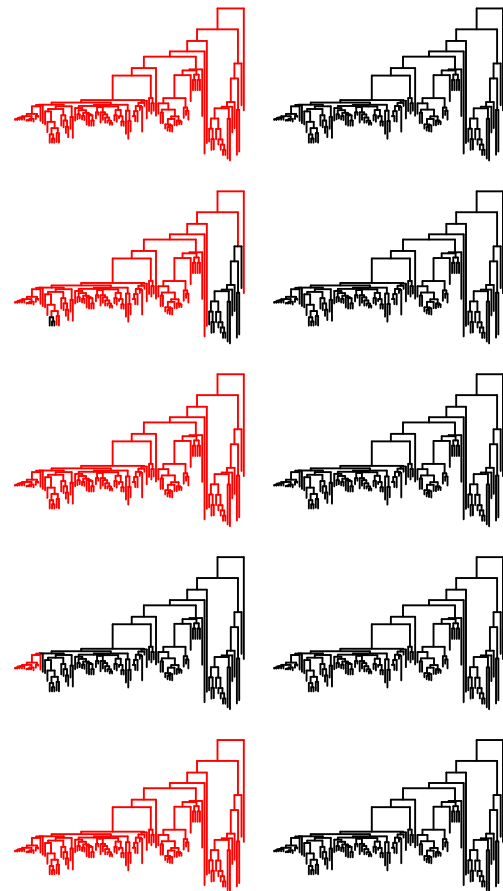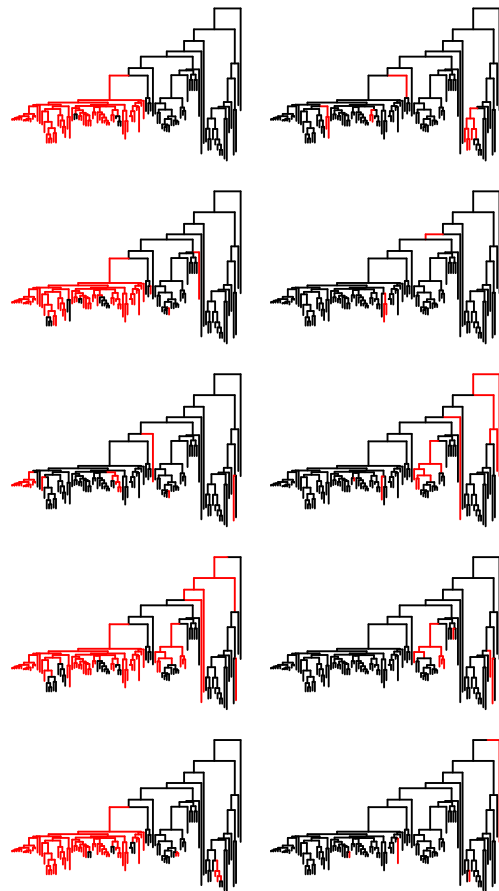

Supplement: S2 Fig — The rates of turnover from a functional state to a non-functional state along the tree for both coding and intergenic sites was taken from Rands et al. [25]. The back-mutation rate from a non-functional to a functional state assumes an equilibrium proportion of functional sequence of 7%. A red branch indicates functional state, a black branch indicates non-functional state. The simulations are conditioned on the human lineage (far left of the tree) being either in the functional state (columns one and three) or the non-functional state (columns 2 and 4). (PDF) [file pgen.1008827.s004.pdf]

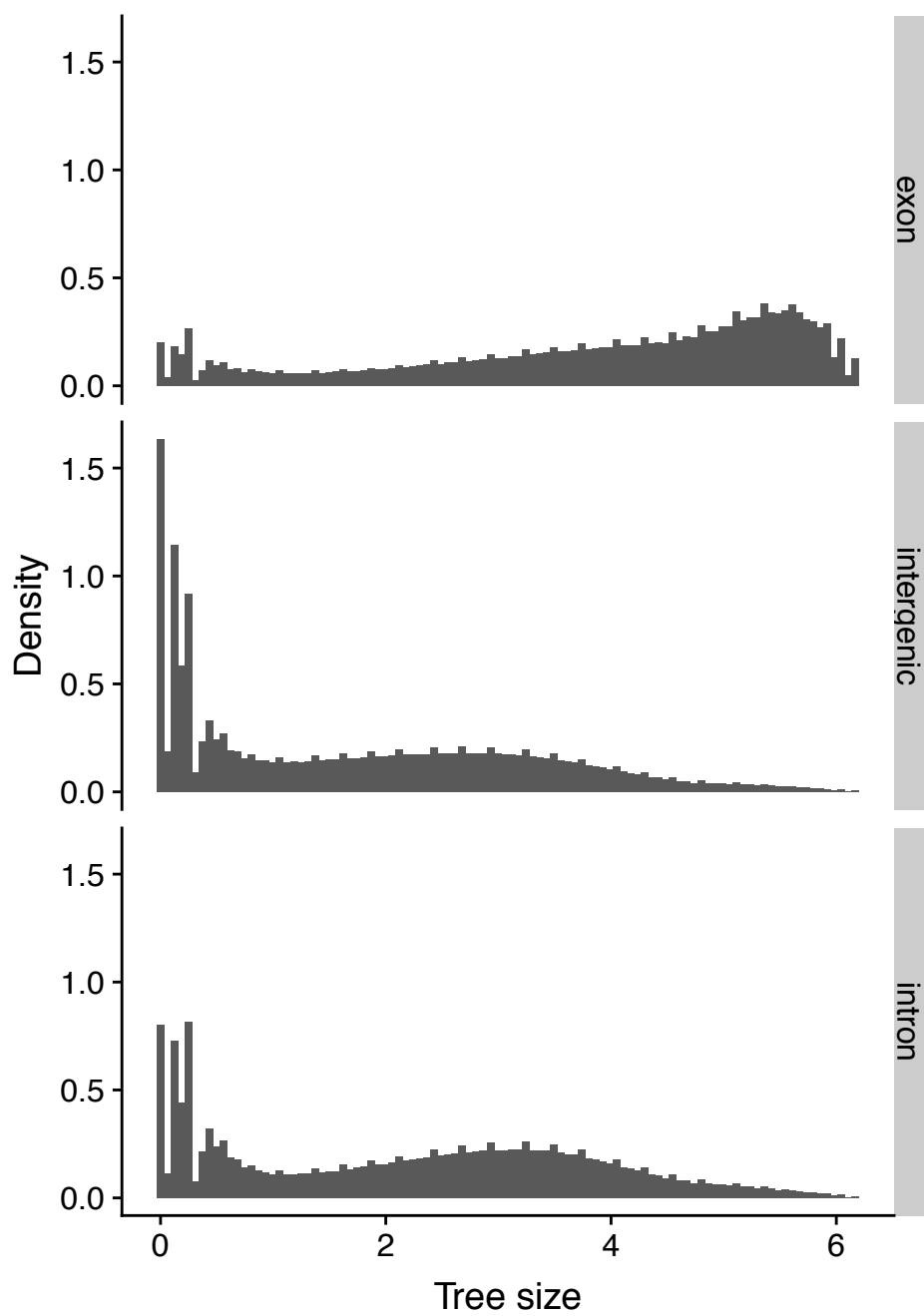

Supplement: S3 Fig — The tree size is in units of expected neutral substitutions on the tree. It is taken from the first column of the GERP/gerpcol software output, which takes into account the reduced tree size due to missing data in some of the species. (PDF) [file pgen.1008827.s005.pdf]

**A**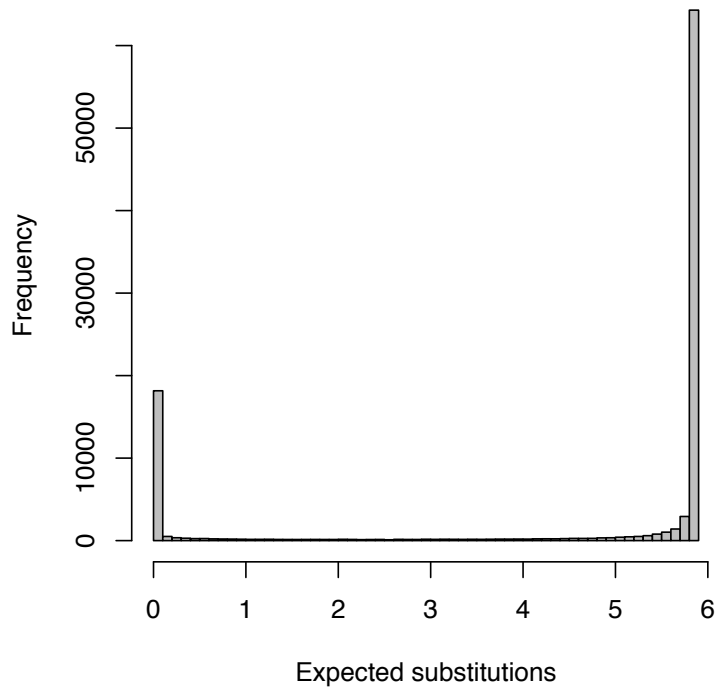**B**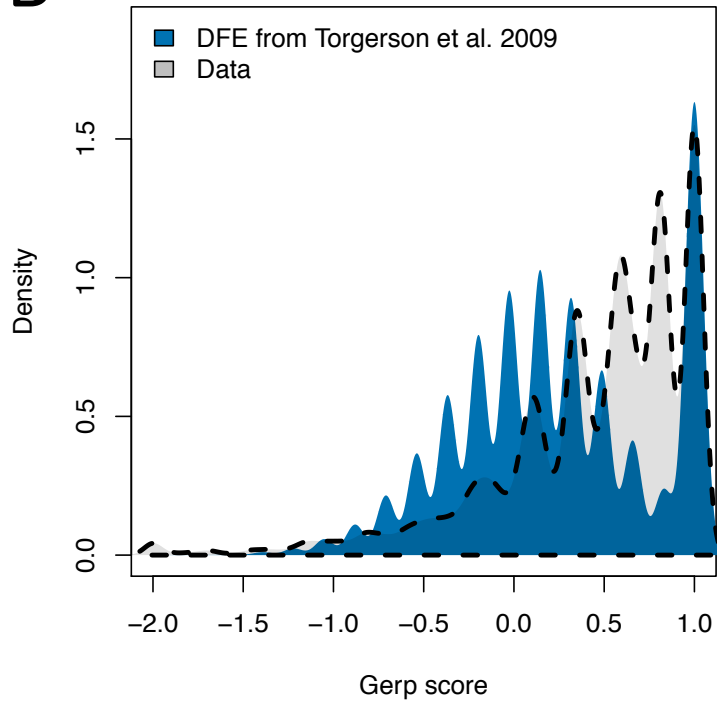

Supplement: S4 Fig — The distribution of expected substitutions (A) given a DFE for noncoding conserved elements from Torgerson et al. [45]. The assumed tree size of 5.85 should reflect almost perfect alignment in the commonly used 36 mammalian species alignment (same as in Fig 4C and 4D). Note that the vast majority of sites either experience an expected substitution rate of neutral sites (peak at 5.85) or zero substitutions. When assuming a Poisson distribution of substitutions on the tree to compute standardized GERP scores (B), the score distribution reflects a mixture of either strongly selected sites (peak at standardized GERP score of one) or a neutral distribution of substitutions (distribution centered at zero). However, the empirical distribution of standardized GERP scores (gray) contains a considerable density of sites with a score between 0.5 and 0.8 that cannot be fit by a model of a DFE estimated for noncoding conserved elements. (PDF) [file pgen.1008827.s006.pdf]

A

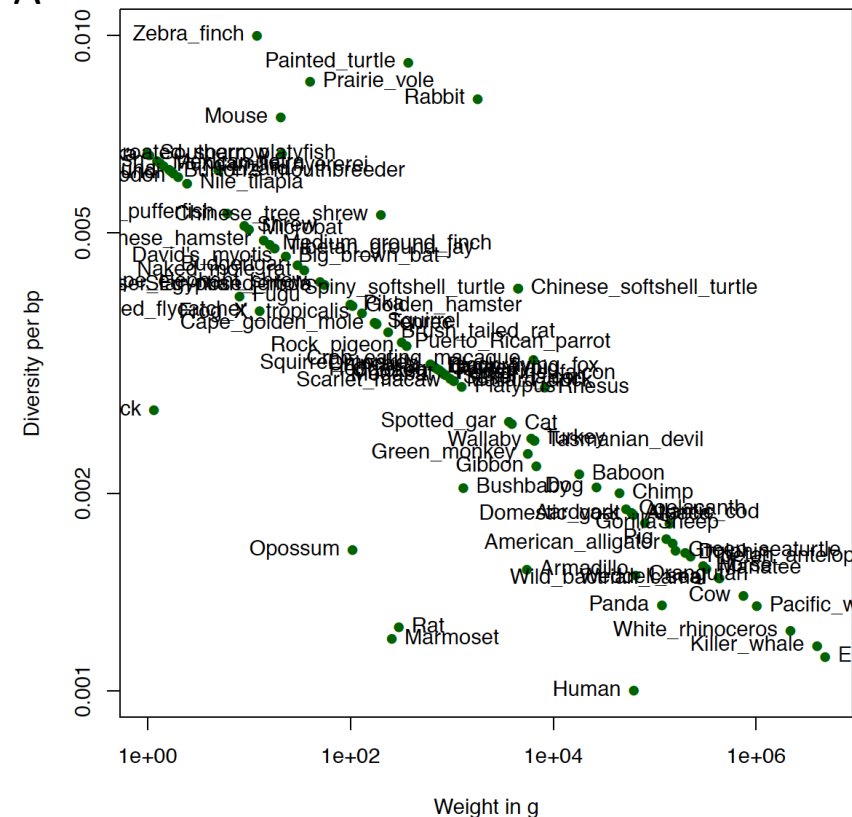

B

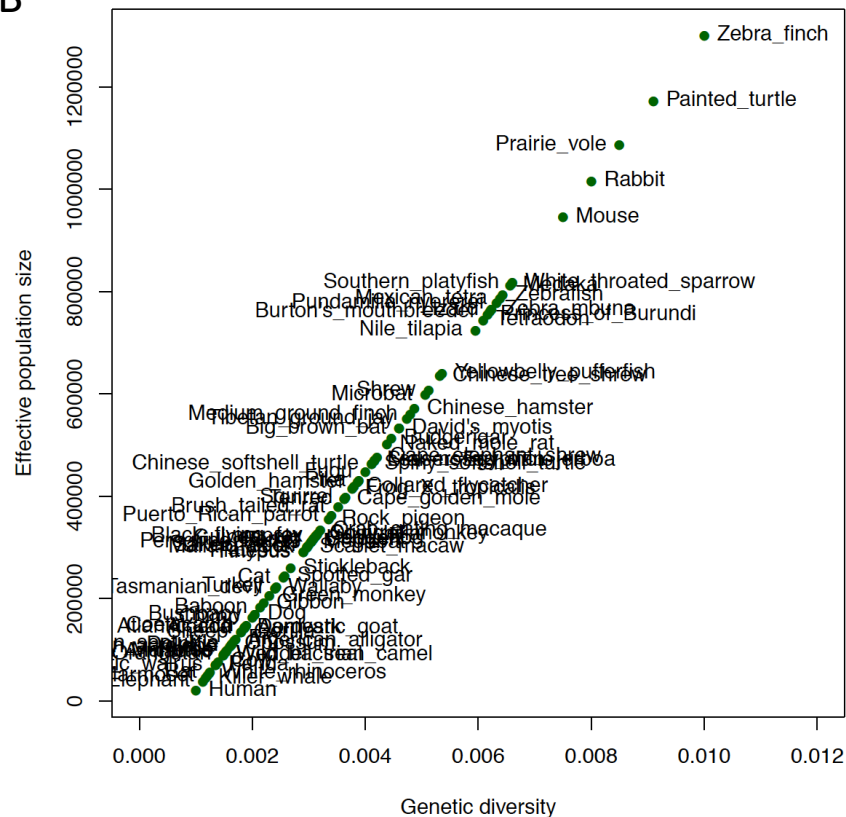

Supplement: S5 Fig — (A) We collected values of synonymous diversity from the literature for a subset of 13 species and predicted synonymous diversity for the remaining 23 species by assuming a linear relationship between log(bodyweight) and log(diversity). (B) Synonymous genetic diversity is then transformed into haploid effective population size by a simple linear interpolation, assuming a haploid population size of humans of 40,000 and of mouse of 1,160,000 (i.e. 20,000 and 580,000 individuals, respectively). (PDF) [file pgen.1008827.s007.pdf]

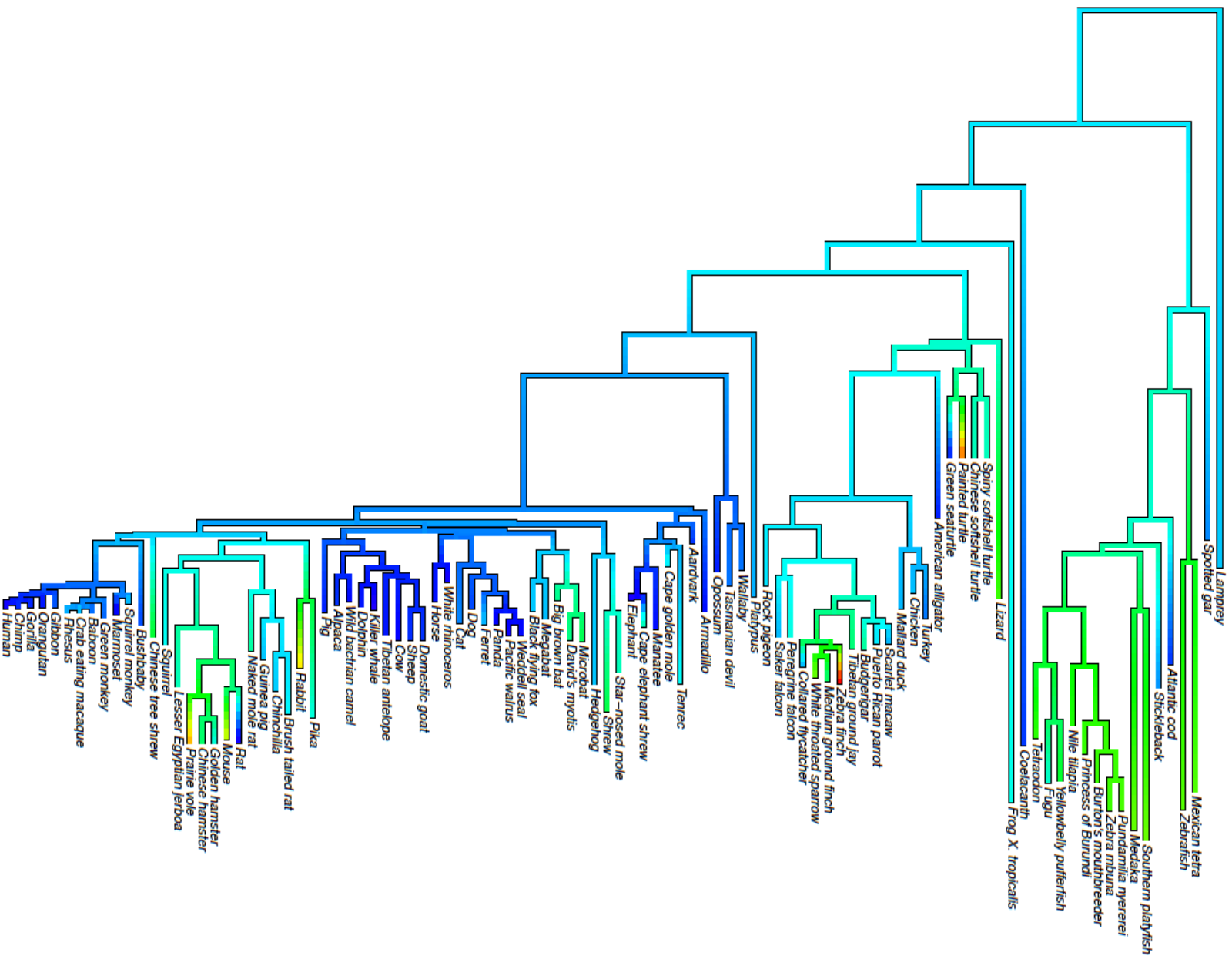

20000

Effective population size

1300000

Supplement: S6 Fig — The effective population size of internal nodes was predicted assuming a Brownian model (see S1 Text). (PDF) [file pgen.1008827.s008.pdf]

**A**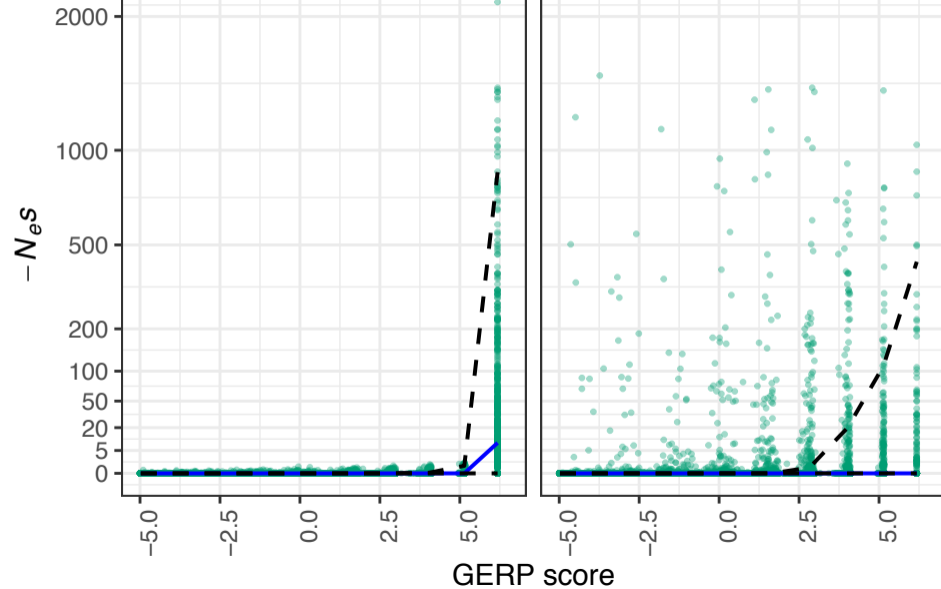**B**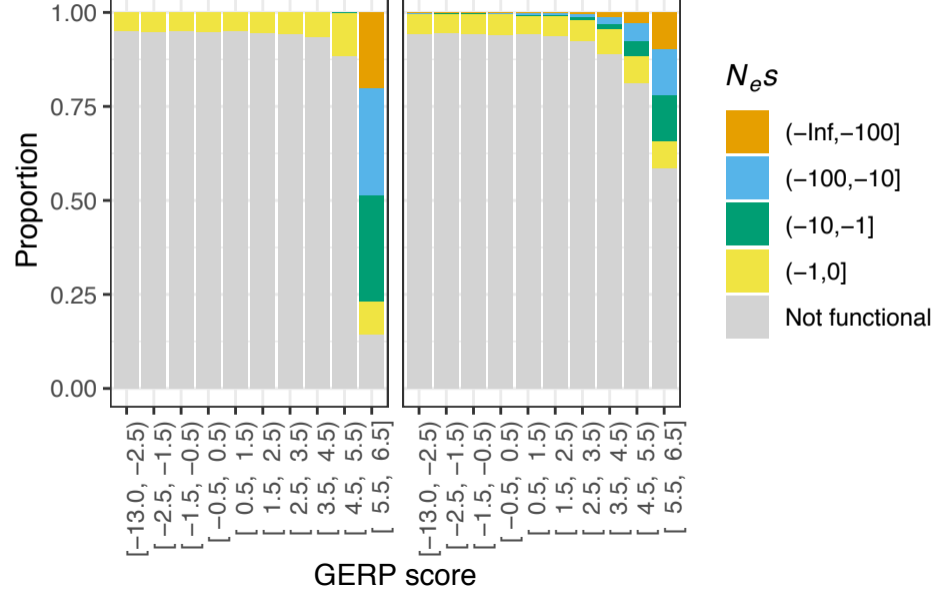

Supplement: S7 Fig — Simulations assume the phylogeny and effective population sizes depicted in S6 Fig. (A) Nes values as a function of GERP scores for a model without turnover of functional sequence across the 36 species tree (left) or where there is turnover modelled according to our Markov model, using the turnover rate from Rands et al. [25] for noncoding elements (right). The blue line represents the median Nes value given a specific GERP score, whereas the dashed lines represent the 2.5% and 97.5% quantiles. (B) Distribution of Nes values for GERP scores when there is no turnover (left) and when there is turnover of functional sequence. Note that when there is turnover, the majority of the sites with high GERP scores (>5.5) are not functional. (PDF) [file pgen.1008827.s009.pdf]

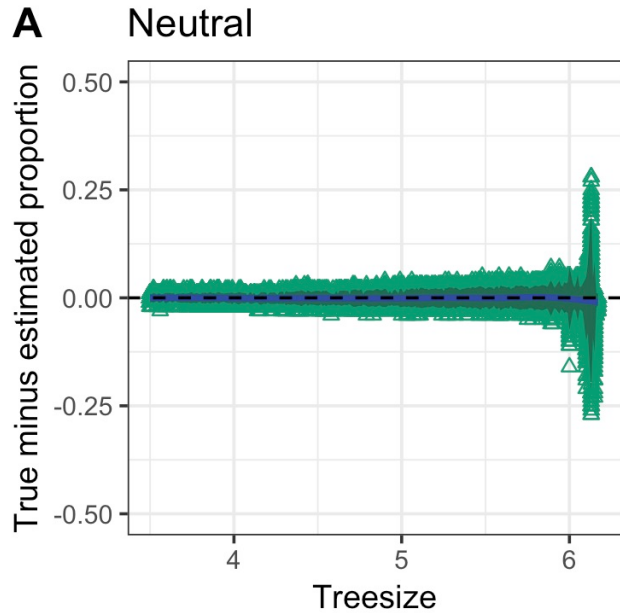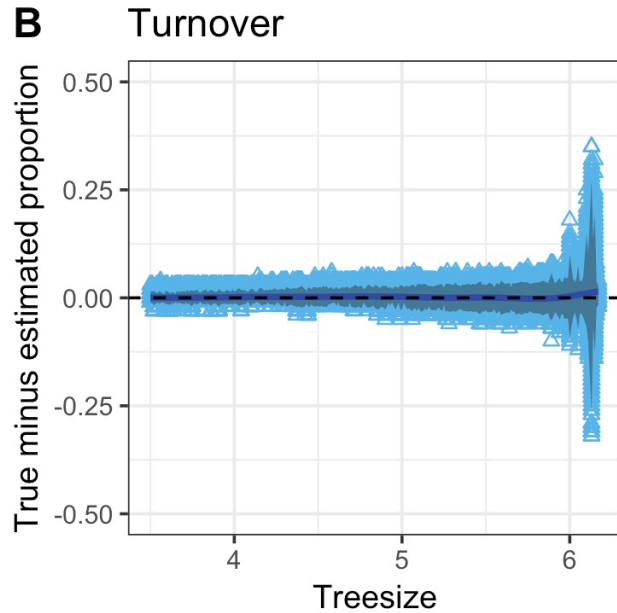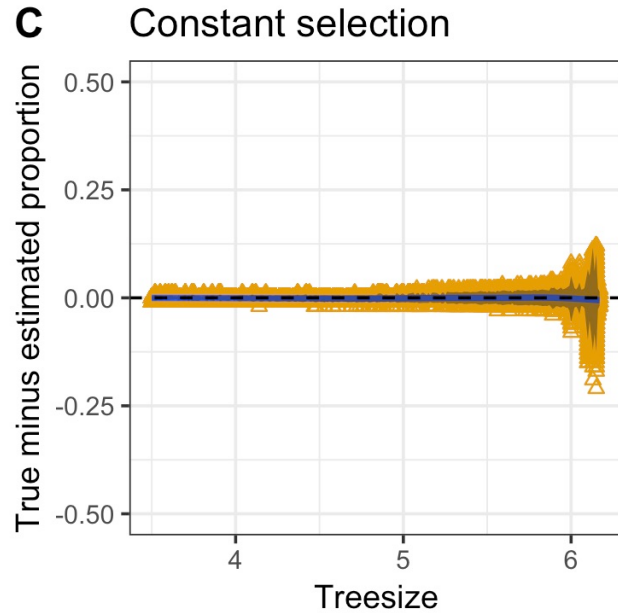

Supplement: S9 Fig — The true minus estimated proportion of neutral sites (A), sites under functional turnover (B) and sites under constant selection (C) were computed using estimates from 500 replicated simulations. For each simulation, the parameters and tree sizes were chosen to reflect the empirical estimates and distribution (Fig 6A and S3 Fig). The gray shaded area reflects the mean +-2 SD. The blue line is a fitted loess curve to the data. It is centered on zero, suggesting that the estimates are unbiased across all tree sizes. Note however that the variance in error increases for tree sizes > 6 because of the small number of sites with alignment across most species. (PDF) [file pgen.1008827.s011.pdf]

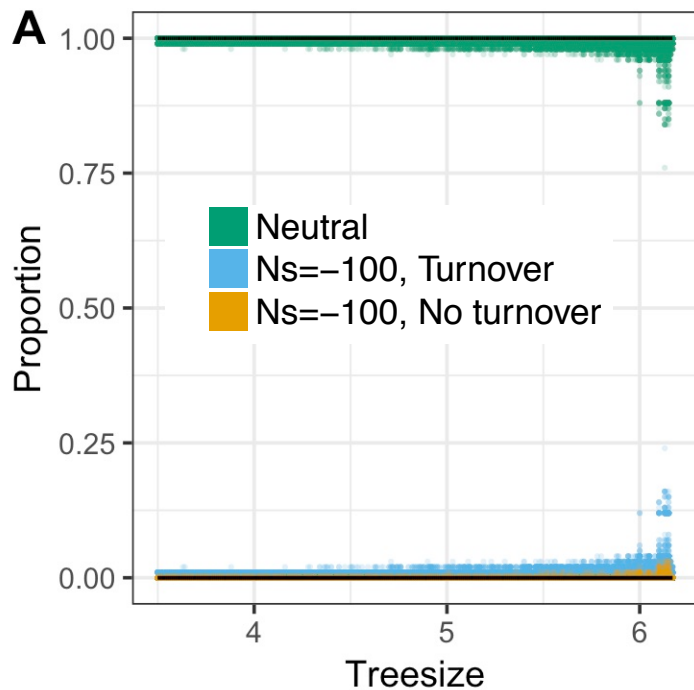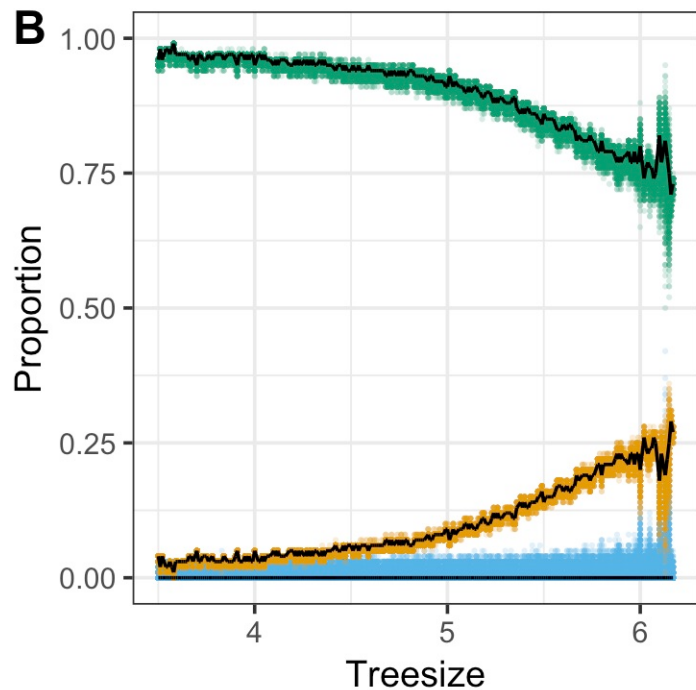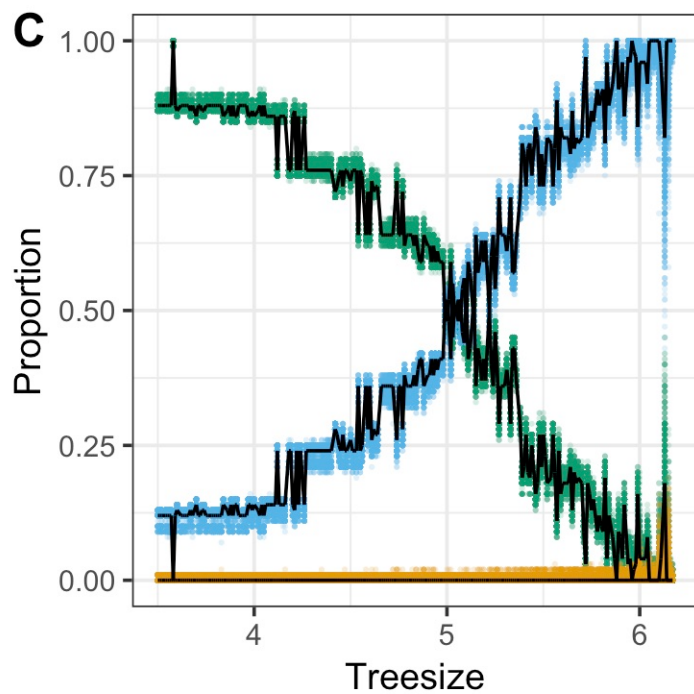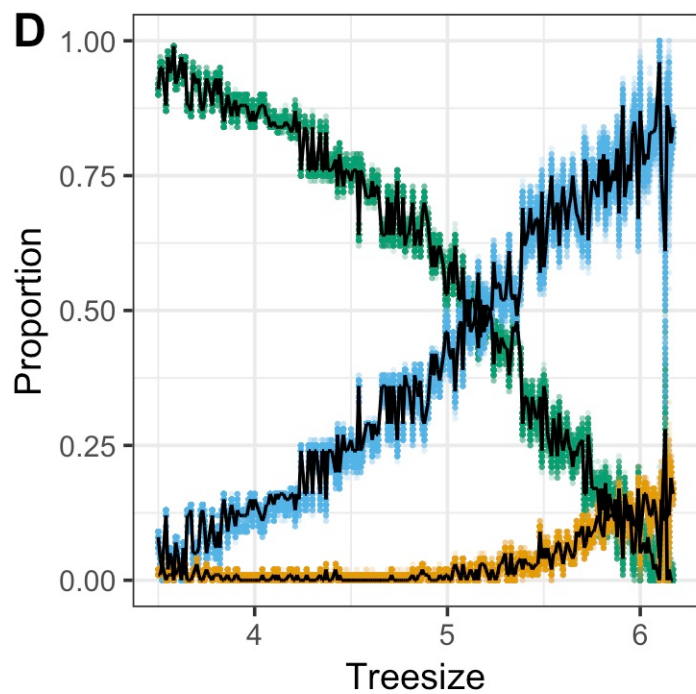

Supplement: S10 Fig — The estimated proportion of three different components of sites: neutral sites (N; green), sites under functional turnover (TO; blue) and sites under constant selection (C; orange), estimated from 500 replicated simulations. For each simulation, the parameters and tree sizes were chosen to reflect the empirical estimates assuming four different models: (A) only N sites, (B) mixture of N and C sites, (C) mixture of N and TO sites, and (D) mixture of N, TO, and C sites. The empirical (i.e. true) proportions are plotted as a black line. For most tree sizes the number of sites is large enough to reliably estimate the proportion of components under the four mixture models. However, the variance in error increases for tree sizes > 6 because of the small number of sites with alignment across most species. (PDF) [file pgen.1008827.s012.pdf]

**A**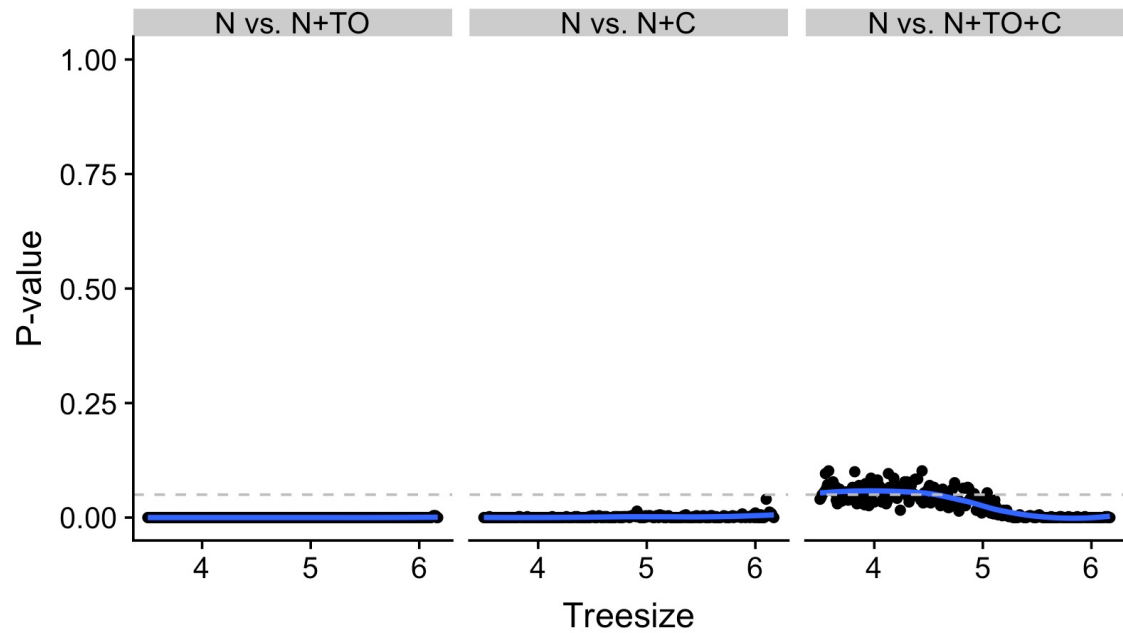**B**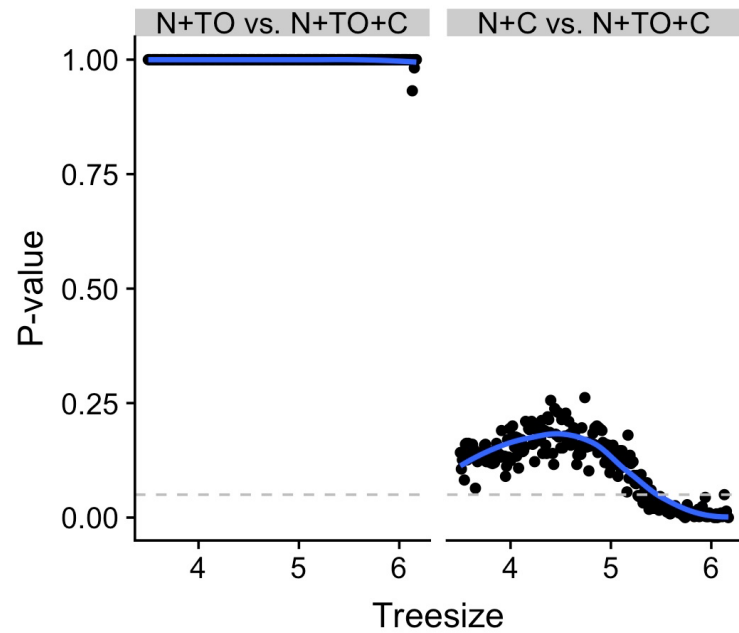

Supplement: S11 Fig — The points denote p-values of rejecting the null hypothesis for each tree size. The p-value is calculated by comparing a null distribution of the test statistic Λ with the value of Λ observed in the data, where the test statistic Λ is comparing the fit to the data of a more complicated model with more components with the fit of a simpler model with fewer components (see Methods). The null model and the alternative model are indicated at the top of each plot. The null distribution of Λ was calculated from 500 simulations under the respective null model. The null model is assuming only neutral sites (A), neutral plus turnover sites (B, left column), or neutral plus constantly selected sites (B, right column). Points below the horizontal dashed line are significant assuming a false positive rate of 5%. The blue line is a smooth loess curve fitted to the points. Note that the data reject a null model of pure neutrality across all tree sizes (p < 0.05). For tree size > 5, the full model (N+C+TO) significantly improves the fit over the N+C model, suggesting a significant role of functional turnover for regions with large tree sizes, i.e. with alignment across most species. (PDF) [file pgen.1008827.s013.pdf]

**A**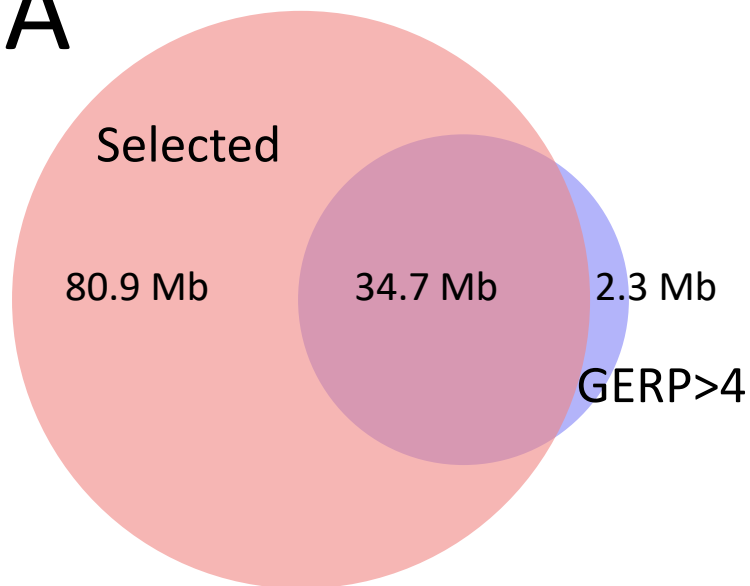**B**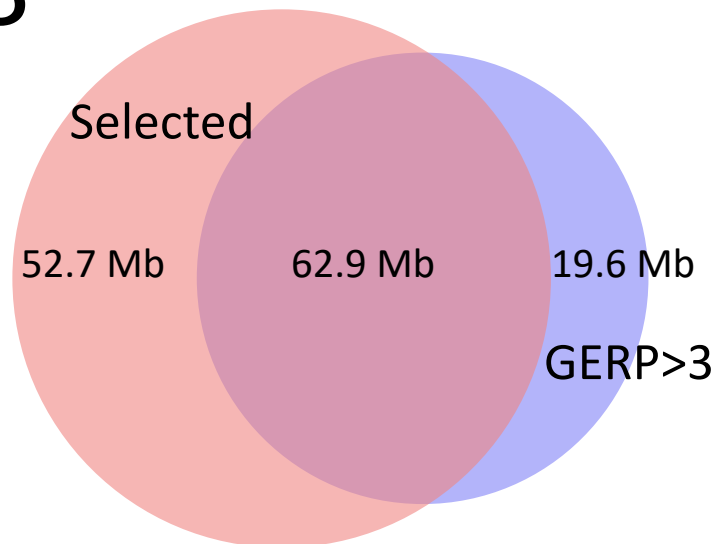**C**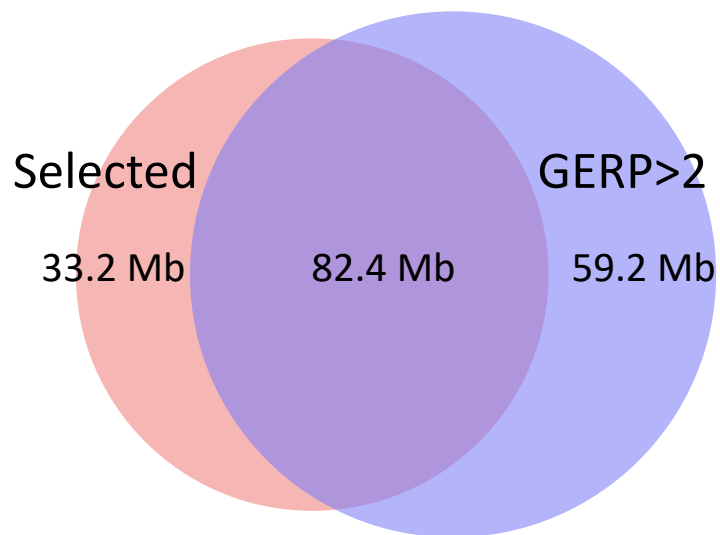**D**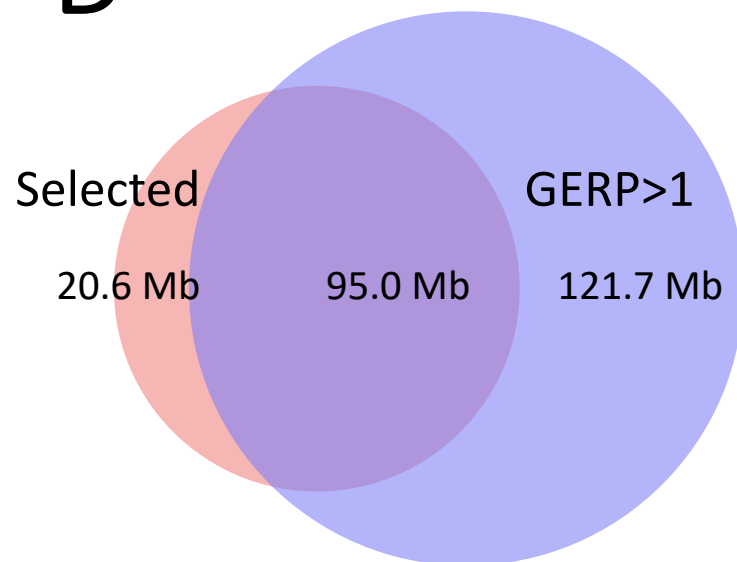

Supplement: S12 Fig — In our estimated full (N+C+TO) model, sites with tree size less than 3.5 are predicted to be exclusively neutral. Thus, we assume that these sites are filtered out accordingly. Blue denotes the number of sites in the human genome with GERP scores: (A) >4, (B)>3, (C) >2, (D) >1. Red denotes the number of sites inferred to be under selection in the human lineage. In C and D, the number of sites with GERP scores >2 or >1 is larger than the number of bases under selection. Note that any filtering strategy based on the GERP score can either have high sensitivity or high specificity, but not both. (PDF) [file pgen.1008827.s014.pdf]

GERP score

Model

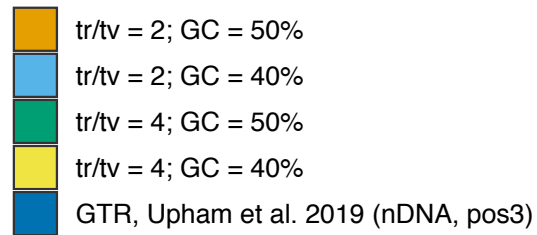

5

0

-5

-10

-8

-7.5

-7

-6.5

-6

-5.5

-5

-4.5

-4

-3.5

-3

-2.5

-2

-1.5

-1

-0.5

0

$N_e s$

Supplement: S13 Fig — Violin plots of simulated GERP scores on a 36 species phylogeny assuming Nes values ranging from 0 to -8 in steps of 0.5. The colors indicate different models of nucleotide evolution. See S1 Text for details. (PDF) [file pgen.1008827.s015.pdf]

**B**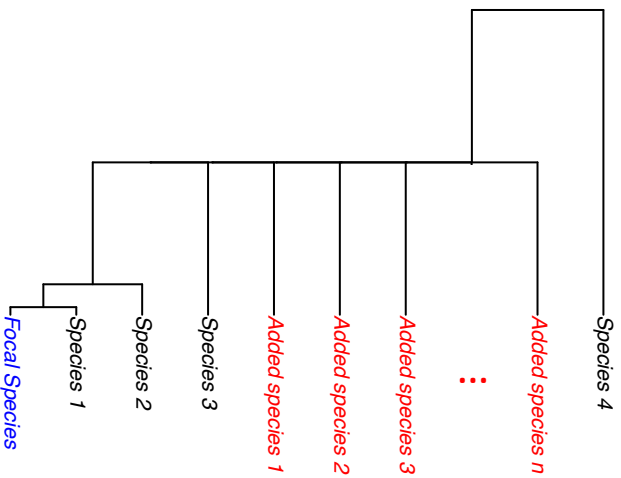**D**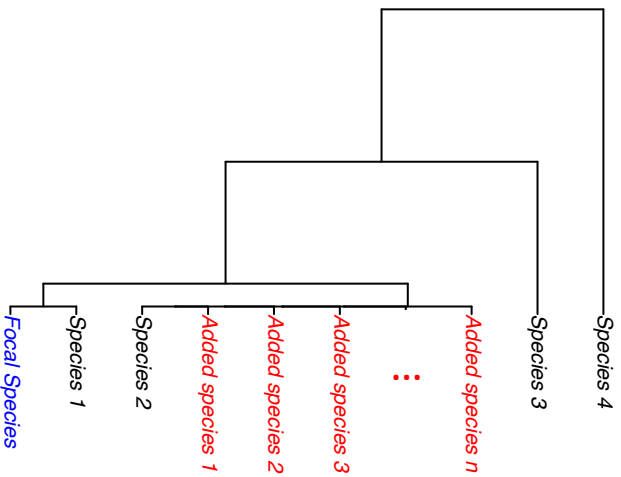**A**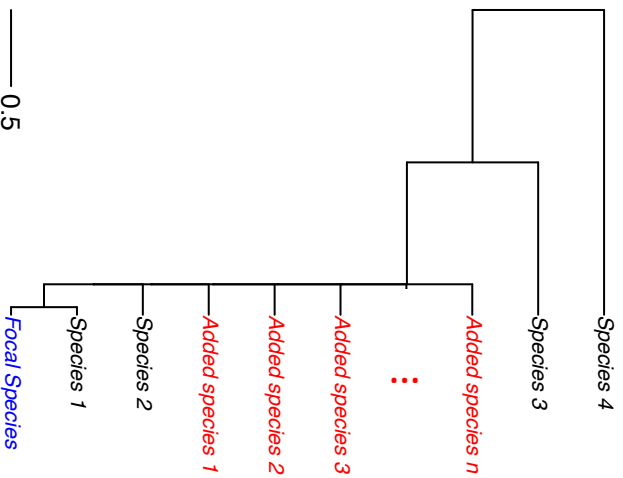**C**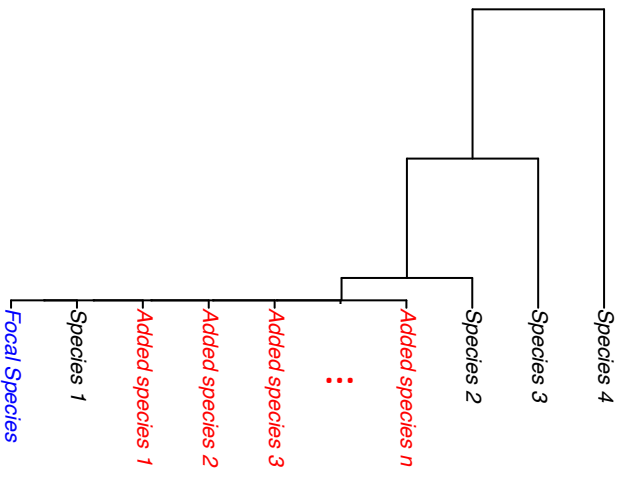

— 0.5

Supplement: S14 Fig — These trees in (A)—(C) represent a primary tree with different degrees of relatedness of a focal species to four other species. The scale of branch lengths in units of subs/site is shown in (A). To investigate the increase in power for detecting functional sites in the focal species, new species are added to this primary tree. The relatedness between the focal species and the added species is 0.4 subs/site (A), 2 subs/site (B), and 0.1 subs/site (C). In (D), every added species is closely related to species 2 (0.1 subs/site) but more distantly related to the focal species (0.4 subs/site). These trees are the basis for the simulations of alignment data and the computation of power for detecting functional sites in S15 Fig. See S2 Text for details. (PDF) [file pgen.1008827.s016.pdf]
