# Supplementary material for: Population genetic models of GERP scores suggest pervasive turnover of constrained sites across mammalian evolution
Source: PLoS Genet. 2020 May 29;16(5):e1008827. doi: 10.1371/journal.pgen.1008827 (PMC7286533; doi:10.1371/journal.pgen.1008827)
Supplement: S8 Fig — The overlap OModel between the probability density of a statistic estimated from the data (blue area) and the probability density of the same statistic estimated under a model (red area) is defined as the area of overlap between the two distributions (striped area). Since probability densities integrate to one, the maximum value of OModel is one if both distributions fully overlap. If the two distributions do not overlap, the OModel is zero. (PDF) [file pgen.1008827.s010.pdf]

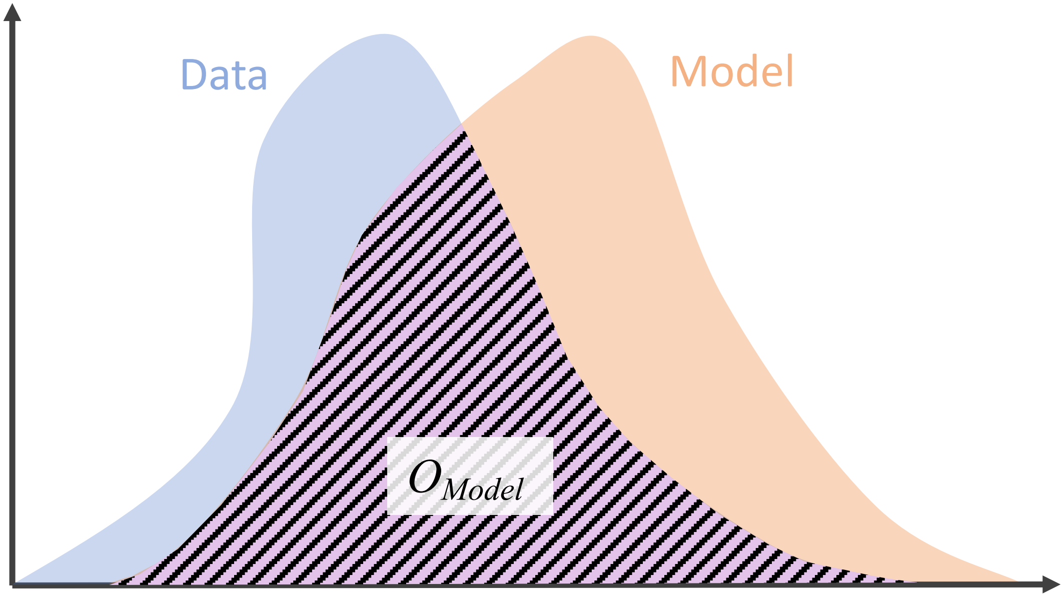
Data
Model
O_{Model}
