# Supplementary material for: Population genetic models of GERP scores suggest pervasive turnover of constrained sites across mammalian evolution
Source: PLoS Genet. 2020 May 29;16(5):e1008827. doi: 10.1371/journal.pgen.1008827 (PMC7286533; doi:10.1371/journal.pgen.1008827)
Supplement: S15 Fig — Power is calculated based on the simulation of substitutions on the trees shown in S14 Fig., assuming different levels of turnover and selection coefficients. Left panels in (A)-(D) show no turnover. Right panels show intergenic levels of turnover with turnover rate as estimated in Rands et al. [25] for noncoding elements. Middle panels show intermediate turnover with a rate half of that in the right panels. See S2 Text for details. (PDF) [file pgen.1008827.s017.pdf]

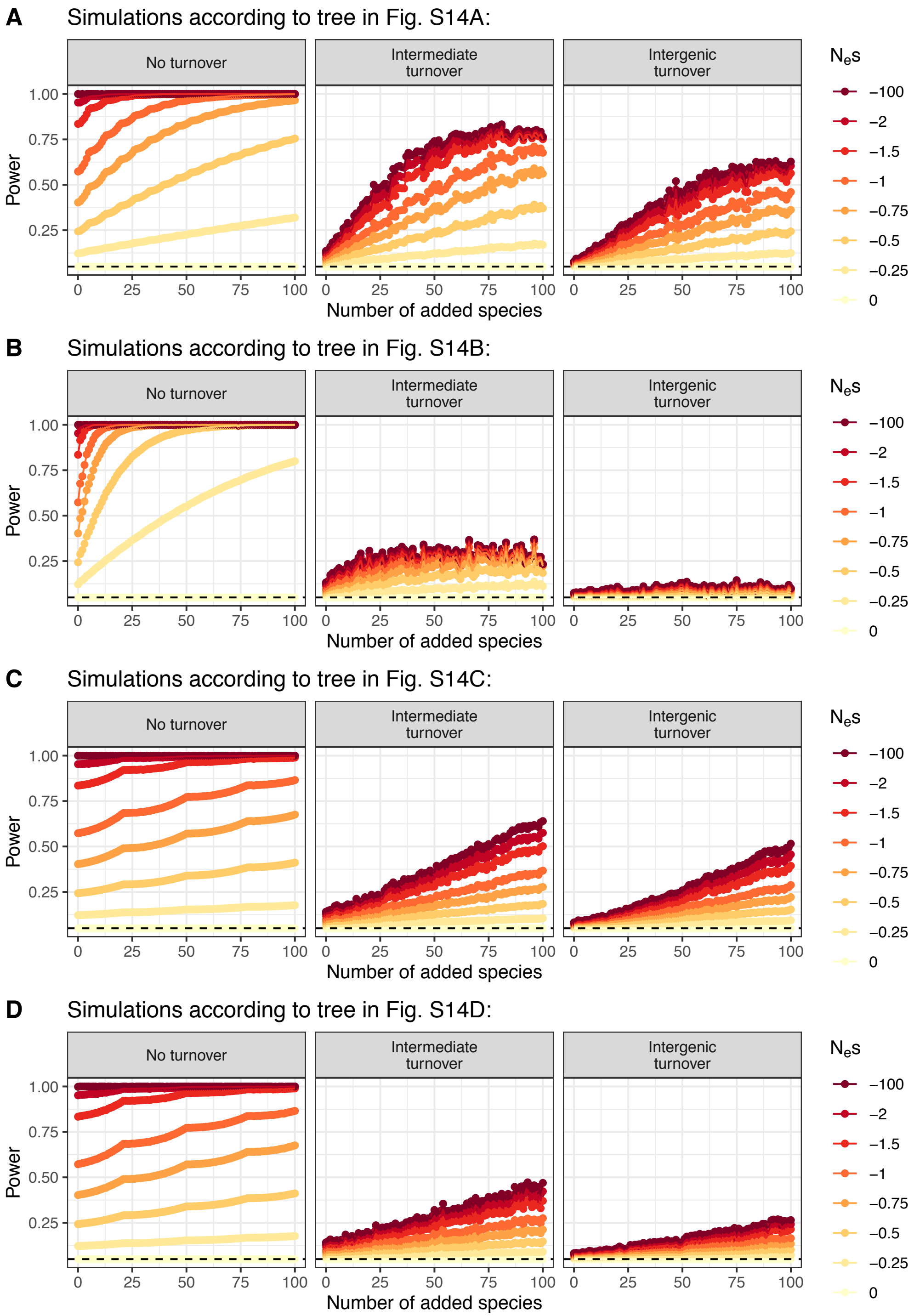
A
Simulations according to tree in Fig. S14A:
B
Simulations according to tree in Fig. S14B:
C
Simulations according to tree in Fig. S14C:
D
Simulations according to tree in Fig. S14D:
No turnover
Intermediate turnover
Intergenic turnover
Power
Number of added species
N_es
−100
−2
−1.5
−1
−0.75
−0.5
−0.25
0
