# Supplementary material for: Population genetic models of GERP scores suggest pervasive turnover of constrained sites across mammalian evolution
Source: PLoS Genet. 2020 May 29;16(5):e1008827. doi: 10.1371/journal.pgen.1008827 (PMC7286533; doi:10.1371/journal.pgen.1008827)
Supplement: Attachment — Submitted filename: GERP paper -- PLOS Genetics response FINAL.pdf [file pgen.1008827.s018.pdf]

'Population genetic models of GERP scores suggest pervasive turnover of constrained sites across mammalian evolution'
Christian D. Huber, Bernard Y. Kim, Kirk E. Lohmueller

Response to reviewers (submitted to PLOS Genetics)
Our responses are provided in red.

Reviewer #1:
GERP conservation scores are often used to evaluate which mutations are deleterious. Using simulations, the authors show that GERP scores do not predict the strength of purifying selection well, turnover of functional elements and lineage-specific constraint complicates the interpretation of GERP scores, and most intermediate GERP scores may be sites affected by turnover. Interestingly, simply increasing the number of species does not uniformly increase power if the simulation is done with turnover. Based on mixture models, the authors estimate that 4.5% of the noncoding genome is under selection in human.

I have only a few comments:

1) In the original paper, the GERP score is simply a measure of how many substitutions have been purged by purifying selection at a particular site. It is a measure of overall constraint, but not a direct measure of how deleterious a particular mutation is. Therefore, the authors should please define how the GERP score of a derived mutation is actually calculated (they refer to this several times in the introduction, e.g. page 6).

We agree with the reviewer that we should address the conceptual gap between the GERP score of a site (which is a measure of conservation across multiple species) and the deleteriousness of a derived mutation that is segregating within a species. We add the following clarification (l. 101-107):

"As such, the GERP score is a measure of sequence conservation across multiple species. However, GERP scores have been commonly used in evolutionary genomic studies as a measure of the strength of selection acting on derived mutations segregating within a single species. In these applications, it is assumed that mutations that appear at sites that are highly conserved across many species are deleterious and thus contribute to genetic load within a species. Quantitatively, for each segregating site within a species the GERP score is assigned to the derived allele segregating at that site."

########
2) This statement "It may be possible to reconcile these estimates by noting that they measure different processes—functional assays assess whether the nucleotide has a biological function, but this function may not necessarily be related to fitness (Graur et al. 2013; Doolittle 2013)". Graur argues that biological function should be applied in the

sense of which function was evolutionarily selected for. Biochemical activity as measured by Encode's 'functional assays' does often not imply a selected function.

We thank the reviewer for making us aware of our inconsistent terminology. We now consistently refer to 'biochemical activity' as any type of biochemical signal that might not necessarily imply a selected function, and we refer to 'biological function' if selection is implied.

We specifically define this in the revised manuscript, lines 151-155, where we write, “*It may be possible to reconcile these estimates by noting that they measure different processes—functional assays assess whether the nucleotide has biochemical activity, but this activity may not necessarily be related to fitness [35,36]. As such, mutations at biochemically active sites may not have an evolutionary impact and thus could appear to be neutral in comparative genomic approaches*”.

########
3) Page 20: Why not using the AIC criterion to evaluate whether added model complexity is justified?

The distribution of GERP scores is irregular and therefore cannot be easily modeled by common probability distributions (Davydov et al. 2010). Thus, we cannot derive the likelihood or the AIC of a model given the data. However, simulations under each examined model allow us to sample from the null distribution of the GERP score. This simulation-based null distribution allows us to test if adding another parameter to the model significantly improves how well the data fits the model, *i.e.* this approach guards against overfitting.

########
4) It would be interesting to explore the effect of adding more closely-related species. E.g. the authors could use the same data generated for Figure 4 but then restrict the analysis on the subtree of primates, where the amount of turnover is expected to be less than in the entire tree.

This is an excellent question and we thank the reviewer for making us think more about the phylogenetic relationship of added species. Our results definitely suggest that power increases when adding closely related species, even in the case of functional turnover. This is reflected in Fig 4 of the revised manuscript by the increase in power from tree size 4 to 6 in the middle and right panel. Because we started with humans as the focal species and then successively added the next most closely related species, the increase in power comes from adding additional primate species.

However, adding only very closely related species might not strongly increase phylogenetic information about the conservedness of sites since the species are expected

to have very similar sequences even under neutrality. For example, the size of the primate subtree in the 100 vertebrate tree used in Fig 4 only has a size of 0.47 subs/site, whereas the full tree has a size of 18.46 subs/site. We refer to this tradeoff in the Discussion (lines 575-580):

"...computing conservation scores from closely related species with a shallow phylogenetic relationship is advantageous since the genomes have a highly correlated functional state and are readily alignable to the focal species. However, if the overall tree size is too small, then conservation (i.e., a lack of substitutions) is harder to detect and power is low. This leads to a tradeoff between tree size and relatedness between the included species (see also S2 Text)."

To explore this topic further, we generated a conceptual model where we add species to a phylogenetic tree and test the power of detecting selection in one focal species (say, humans). We vary the relatedness of the added species to the focal species. When there is turnover, adding species with an intermediate level of relatedness (e.g. equivalent to adding species with divergence to humans similar to mouse, rat, etc.) leads to a large increase in power per added species. There is almost no added value when adding highly diverged species (e.g., adding species with divergence to humans similar to lamprey or zebrafish). If the added species are too closely related, then power increases steadily but only very slowly (e.g., adding primate data such as baboons, macaques, or marmosets). Roughly, the increase in power when adding the mouse sequence is twice the increase in power of adding a primate sequence. Finally, we also note that adding species that are closely related to an already sampled species (e.g. adding mouse data when rat is already included) again does not substantially increase power.

These results are now summarized in a newly added supplementary text (S2 Text) and referenced in the Discussion.

########
5) I believe "or under selection" should be removed from this sentence "For example, approximately 61.6% of GERP scores >5.5 in our simulations are from sites that are not functional or under selection in humans"

We agree that this statement is confusing. We removed "or under selection" from the sentence in the revised manuscript.

Reviewer #2:
Huber et al. examined how one measure for assessing sequences under selection, Genomic Evolutionary Rate Profiling (GERP), is related to the population genetic strength of selection (NeS). They found: (1) they are related, (2) GERP distribution is impacted by changes in selection coefficient, or function over time, (3) more turnover in sequence elements' functions correlates with smaller optimal tree size, (4) 4.5% of non-coding human genome is under purifying selection experiencing changes in selection coefficient over the course of mammalian evolution. One major question I have is with regard to the validity of simulation results in reflecting the mammalian evolutionary trajectory. I also have other questions that I hope the authors may find useful. This is a very long paper and I may have missed some of the points – but the authors may consider this point as a reader' perspective – possibility of tightening the manuscript up some.

We thank reviewer 2 for their comments, which greatly helped us strengthen the validity of our simulation results. Further, we tightened up the manuscript by moving several results related to the robustness of our inferences to violations of modeling assumptions into a separate SI text (S1 Text). Although we think these results are an important contribution to the field, they are only relevant for a specialized audience and are not necessary for understanding the main results of the paper.

########
1) For reader not familiar with the approach, the abstract's mention of trees would be cryptic – need to provide info on the relevance. Also, spell out GERP in abstract.

We thank the reviewer for making us aware that mentioning trees in the Abstract without further explanation might confuse the reader. We decided to remove any reference to a tree or tree size from the Abstract and instead write, "*Further, we show that for functional elements that have a high turnover rate, adding more species to the analysis does not necessarily increase statistical power*".

We now spell out GERP in the Abstract of the revised manuscript.

########
2) p.5, the authors commented on the use of conservation as a way to detect selection to be "[a] concept [that] given rise to the field of comparative genomics". This is rather inaccurate, as comparative genomics is broader than looking at conservation and certainly the early evolution of the field was contributed heavily by non-evolutionary biologists.

We agree and thank the reviewer for pointing out that this sentence did not adequately capture the role of sequence conservation in comparative genomics. Thus, we have removed this sentence from the revised manuscript.

########
3) p.5, “A number of statistical approaches have been developed to find these sites … showing conservation” – provide citations.

We now added citations to a range of papers that developed or compared statistical methods for the analysis of sequence conservation (Cooper 2005; Pollard et al. 2010; Siepel et al. 2005; Margulies et al. 2003; Asthana et al. 2007; Boffelli et al. 2003; Miller et al. 2004; Mouse Genome Sequencing Consortium 2002).

########
4) p.6, “GERP scores may not provide quantitative evidence of the strength of selection because any deleterious mutations that have a scaled selection coefficient of Nes < -2 will not accumulate as substitutions (Figure 1)” – this is intro section - perhaps talked about this as something that has not been evaluated and put the info in Figure 1 into results? Also, Figure 1A seems show a negative correlation. Will be helpful for the author to provide the GERP scores as distributions (e.g. violin plots) as it is hard to gauge what the central tendency of GERP values is.

We thank the reviewer for calling to our attention the reference to results in the Introduction of the previous version of the manuscript. Since this is not a novel result but derives from known formulas for the probability and rate of fixation of a newly arising deleterious mutation, we replaced the reference to Fig 1 with references to previously published work (Kimura 1962; Lanfear, Kokko, and Eyre-Walker 2014; Lawrie and Petrov 2014; Nielsen and Yang 2003).

We agree that showing the GERP score distribution in Fig 1 as violin plots better illustrate how the GERP scores change with different scaled selection coefficients. We have now done this in the revised manuscript (see the revised Fig 1 below).

Finally, we decided to remove the curve of "Substitution rate relative to neutral" from Fig 1A since this information is not essential here to understand the relationship between GERP and $N_e s$, and a plot with two different *y*-axis labels may confuse the reader.

Please see the revised Fig 1 below.

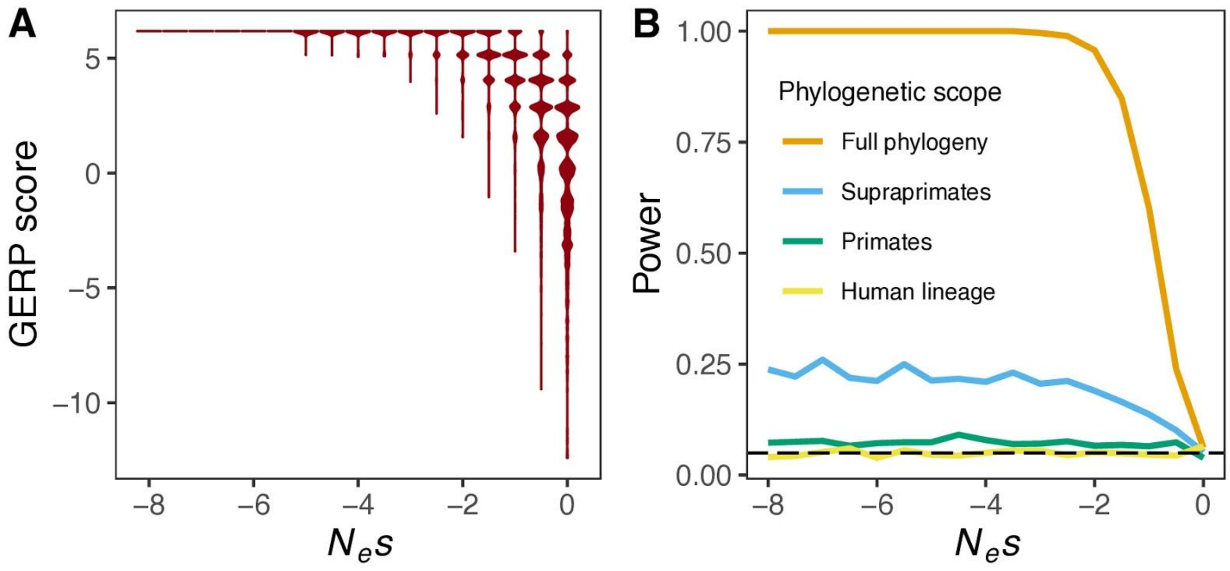

########
5) p.7, “Comparative genomic approaches assume that selective pressures have remained relative stable…” – it will be helpful to point out specific examples here, particularly using GERP as an example.

Here we had intended to say that conservation score methods have poor power for detecting individual nucleotides when selection is confined to a specific subtree. This was shown by Pollard et al. (2010), where, as an example, selection was restricted to a subtree of 14 primate species and power dropped substantially. Thus, these comparative genomic methods assume that selection had consistently purged mutations during most of the phylogenetic history. We try to make this point clearer in the revised manuscript and now cite the key study by Pollard et al. (2010) in lines 135-139:
"Second, most conservation-detection methods assume constant selection pressures across all branches of a phylogeny (Pollard et al. 2010). Any sort of lineage-specific selection, or turnover of functional sequence (i.e. a sequence has a specific regulatory role in one lineage, but does not in another lineage), could potentially be missed by these comparative genomic approaches."

########
6) p.8, “mutations at functional sites may not have an evolutionary impact and thus could appear to be neutral in comparative genomic approaches” – The authors need to be clear here. Given the interests of the manuscript is about detecting selection, it is not clear to me how these “functional” sites that have no evolutionary impact is relevant. Also, in this paragraph, the authors used the word “function” inconsistently. E.g. in the sentence following “sequences may have a biological function in some species and not others…” – here it seems that the “biological function” here is under selection, contradict with the

statement "… whether the nucleotide has a biological function, but this function may not necessarily be related to fitness". This is rather confusing.

We thank the reviewer for making us aware of our inconsistent terminology. We now consistently refer to 'biochemical activity' as any type of biochemical signal that might not necessarily imply a selected function, and we refer to 'biological function' if selection is implied. This terminology was also suggested by other authors (e.g. Graur et al. 2013).

We change the text of the revised manuscript on lines 151-155 accordingly:
"It may be possible to reconcile these estimates by noting that they measure different processes—functional assays assess whether the nucleotide has biochemical activity, but this activity may not necessarily be related to fitness (Graur et al. 2013; Doolittle 2013). As such, mutations at biochemically active sites may not have an evolutionary impact and thus could appear to be neutral in comparative genomic approaches."

Further, we absolutely agree with the reviewer that the interest of our manuscript is about detecting selection, not about detecting biochemically active sequences. However, biochemical assays conducted by the ENCODE project have been interpreted to suggest that a large proportion (80%) of the genome is "functional". Thus, we feel the need to distinguish our definition of biological functionality, which is based on signatures of purifying selection, from a purely biochemically-based definition.

########
7) Figure 1 – I assume the approach for generate the result is based on the simulation of deleterious substitution along a phylogenetic tree discussed in p.30. The authors used HKY85, transition/transversion ratio of 2, and assume equilibrium frequencies to be equal for all four nucleotides. For the first two, would be helpful to know why they were picked and whether some parameter sweep was done to help with picking them out. For the equal equilibrium nucleotide frequency, given the human genome has a mean GC content of 41%, it seems unrealistic – can the authors explain why it was used? How do all these impact the discussion relevant to figure 1? How realistic is the resulted deleterious substitution simulation?

We thank the reviewer for bringing up the very valid point of the robustness of our results to violations of model assumptions. In the simulations for Fig 1, we indeed assume the HKY85 model, a transition/transversion (tr/tv) ratio of 2, and equal equilibrium frequencies. We think that our results are robust to these assumptions for two reasons. First, the GERP software estimates the tr/tv ratio and the nucleotide frequencies from the data, assuming an HKY85 model. The estimated GERP scores should thus be robust to the levels of tr/tv ratio and nucleotide frequencies. Second, the original GERP paper suggests that alternative realistic nucleotide evolution models (*i.e.* different from HKY85) have only negligible impacts on the estimated GERP scores (Cooper et al. 2005).

However, we now further test the robustness of GERP by simulating data under different GC content and mutation models. In previous simulations, we assumed a tr/tv ratio of 2, as estimated for intergenic human data (Bainbridge et al. 2011). However, the tr/tv ratio for genes is quite consistently estimated to be ~4 across multiple mammalian lineages (Rosenberg, Subramanian, and Kumar 2003). Further, the GC content of mammalian genomes varies between 40% and 50% (Romiguier et al. 2010). We thus simulated data under various combinations of tr/tv ratio (2 vs. 4) and GC content (40% vs. 50%). The simulations result in GERP score distributions that are very similar to the one in Fig 1A (See the figure below, which is now presented in S13 Fig of the supplementary materials). Finally, we find similar results when simulating data under a GTR model with parameters estimated for mammals in Upham et al. (2019). We thus conclude that our results are robust to the assumptions of the mutational model.

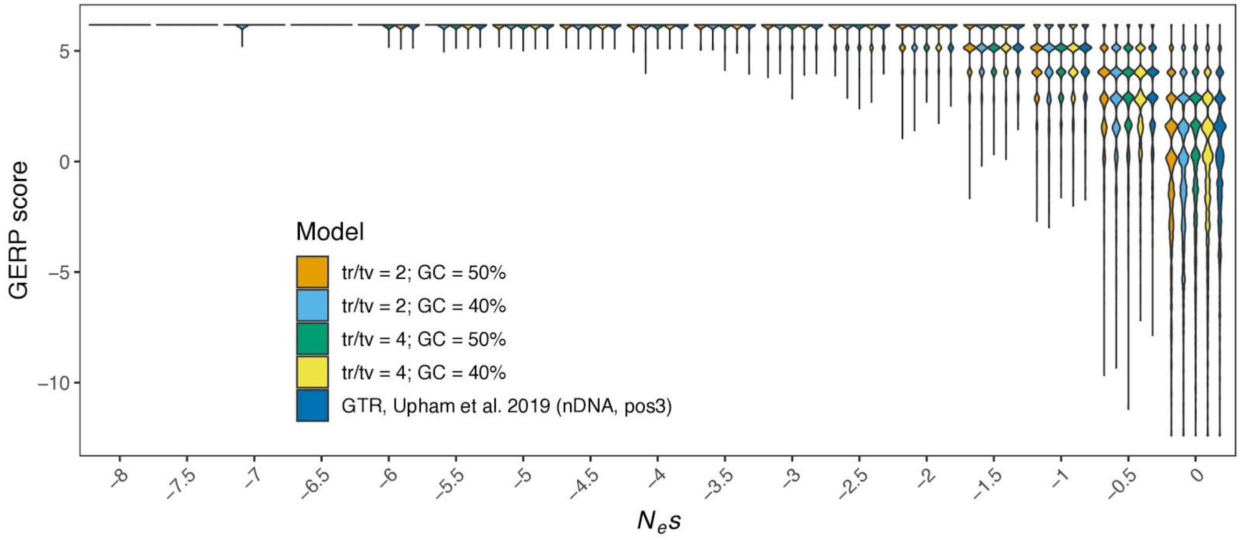

The simulation of deleterious substitutions follows the framework developed in (Nielsen and Yang 2003). It assumes that there is no interference in the fixation process of multiple mutations at different sites, that there are never more than two alleles segregating at the same nucleotide sites, and that the selection coefficient acting on new mutations at a site is constant in a particular lineage. These assumptions are most likely valid in all organisms that we consider, in particular when considering deleterious mutations (Nielsen and Yang 2003).

We now added all these considerations regarding the robustness of our results to the supplementary material (SI text 1) of the revised manuscript.

########

8) p.10, “positive GERP scores are not very predictive of the strength of purifying selection on a particular variant” – this is based on Fig 1A, but the interpretation is problematic. It is true that a GERP score > 4 can be generated by nearly neutral

mutations – the question is how often this happens. It is hard to tell from Figure 1A. It would have been helpful to know the percentile values. Besides, this is assuming that the deleterious simulation is realistic – which the authors should provide arguments that it is.

We changed Fig 1A to better display the distribution of GERP scores for different scaled selection coefficients using violin plots. We agree that strongly selected mutations can be distinguished from nearly neutral and neutral mutations to some degree. However, our point here is that a large range of $N_e s$ values leads to the same maximal GERP score. We try to make this argument more compelling by choosing different $N_e s$ and GERP values, see lines 201-207 in the revised manuscript:
"Fig 1A shows that the largest GERP score (GERP = 6.18, i.e. zero predicted substitutions) can be generated by weakly deleterious mutations as well as very strongly deleterious mutations. For example, both sites with weakly deleterious mutations (e.g., $N_e s$ = -4) and sites with strongly deleterious mutations (e.g., $N_e s$ = -1000) lead to the largest GERP score with high probability (98.2% and 100%, respectively). Thus, observing the largest possible GERP score for a given alignment is not very predictive of the strength of purifying selection."

########
9) Would it make more sense to infer GERP scores using simulated sequence data so the results are more directly comparable with the population genetic parameters used to generated the simulated sequences?

We relate the population genetic parameters of our model ($N_e s$) to the number of substitutions on the phylogeny using the fixation probability of deleterious mutations (Nielsen and Yang 2003). This is a standard approach to inferring selection using divergence data (Yang 1997, 2007) and for simulating genetic data along a phylogeny (Spielman and Wilke 2015). Thus, in our simulations, we model selection via its effect on reducing the substitution rate of deleterious mutations using continuous-time Markov models of sequence evolution. We then compute the GERP score from the simulated alignment data using the GERP++ software package (Davydov et al. 2010). Modelling selection more explicitly via differential fitness among individuals in population genetic forward-in-time simulations (using SLiM or other software packages) is highly computationally impractical (potentially impossible) for the timescales used here.

However, we now provide further discussion of the assumptions behind our use of the Nielsen and Yang framework to simulate sequence data in the supplementary material (S1 Text) of the revised manuscript. See also our response to comment 7, suggesting that our simulation approach is valid for all the organisms that we consider, in particular when simulating deleterious mutations (Nielsen and Yang, 2003). Thus, our model mimics the salient features of the generative model and we believe there is little to be gained from simulating additional sequence data.

########
10) Figure 2: instead of average, can the authors show median values (or violin/box plots) so one can get a better sense of the distribution? Can the authors use color scheme other than red-green for accessibility reason?

To better illustrate the center and spread of the $N_e s$ distribution given certain GERP scores, we added a blue line for the median and two dashed black lines for the 2.5% and 97.5% quantiles. We further thank the reviewer for suggesting we use a color scheme other than red-green. We now use a colorblind-friendly palette in the revised manuscript. Please see the revised Fig 2 and 3 below.

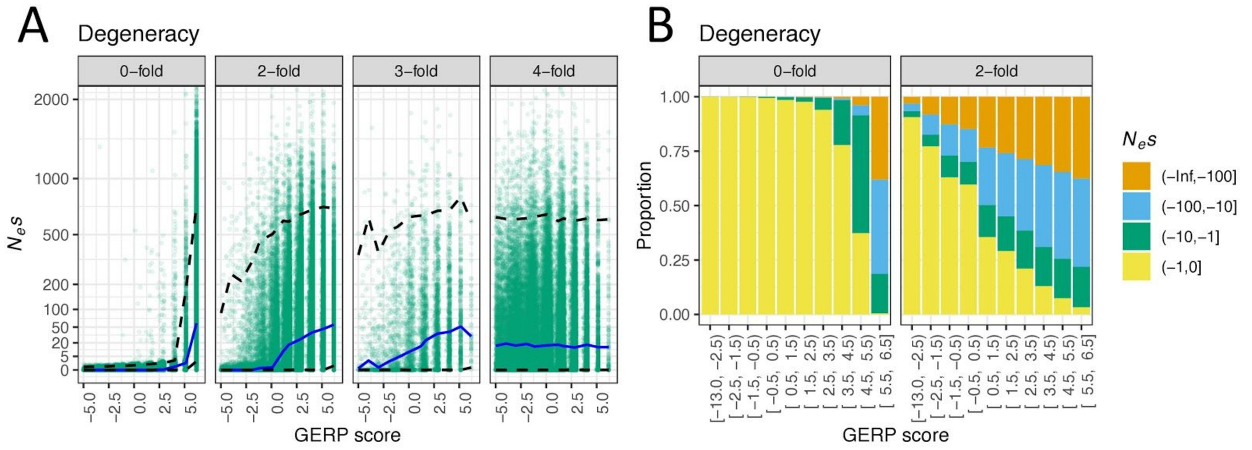

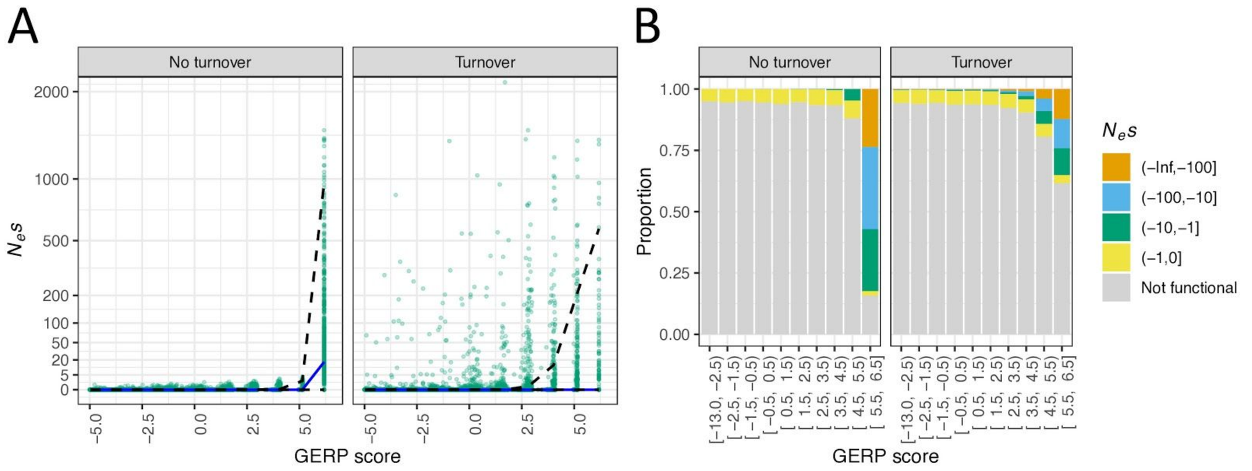

########
11) NeS and NS are used interchangeably in figures, which can be confusing.

Apologies for missing this error. We now consistently use $N_e s$ in all figures.

########
12) p.12, “Many of the deleterious mutations show GERP score ….” – provide exact number, proportion to support claims.

We now do this. We write (lines 236-238), "*At 3-fold degenerate sites, as much as 21% of the sites with $N_e s$ < -1 show GERP scores that are <0 as a result of neutral changes occurring at these sites throughout the evolutionary history of the phylogeny*".
And further below, related to 4-fold degenerate sites (lines 244-245): "*...the average strength of selection is unrelated to the GERP score (Spearman's rho = 0.0061, p=0.16).*"

########
13) p.14, "Assuming functional turnover as outlined … results in a very different pattern" – looking at Figure 3, it is not clear if it would support that the pattern is "very different". Although with turnover there is a lot more scatter, it seems that they can be attributed to outliers (as suggested by Figure 3B and the average line in Figure 3A). The authors discussed the most extreme situation (GERP>5.5) to say that ~62% are not "under selection" – but the criterion for calling selection (e.g. an NeS threshold) was not provided or justified.

We agree that the adverb *very* might be too strong here. We thus removed it and say that assuming functional turnover "*results in a different pattern*". However, for the purpose of using the GERP scores to infer selection pressure in the human lineage, the difference between the constant selection and turnover model is quite relevant. Under the turnover model, many of the sites that are strongly selected in the human lineage have GERP scores that are not distinctive from the neutral GERP distribution. To make this point clearer, we now changed lines 277-285 to say: "*For example, approximately 61.6% of GERP scores >5.5 in our simulations are from sites that are not functional in humans, i.e. mutations segregating at those sites in humans would be neutral, but the GERP score at those sites would strongly signify selection. For comparison, in a model without turnover, only 15.9% of mutations at sites with a GERP score >5.5 are neutrally evolving in humans. Less extreme GERP score cutoffs have a larger proportion of neutral sites even under the model without functional turnover (e.g., 76.0% neutral sites for GERP score >4), which only worsens under the turnover model (85.1%). Thus, even a small amount of functional turnover can dramatically limit the utility of GERP scores at detecting mutations under selection.*"

########
14) p.15 and on, on optimal tree size: One naïve notion in my mind is that, for functional turnover to have less impact on GERP, adding more species, and thus more resolution from a phylogenetic sense, would allow the approach to detect branches with turnover more readily. But the results in Figure 4 demonstrated otherwise. Wonder if the authors can explain this a bit.

The GERP approach assumes constant selection pressures across all branches of a phylogeny, i.e. it does not try to detect branches with turnover. Further, we are specifically interested here in detecting a site that is under selection in a specific target species (in

this case humans), although it might be under selection in many other species as well. Under the model of functional turnover, adding many branches from unrelated species where the specific site might not be under selection (due to turnover) adds random noise to the GERP score and thus reduces power for detecting selection in the target species.

To explore this topic further, we generated a conceptual model where we add species to a phylogenetic tree and test the power of detecting selection in one focal species (say, humans). We vary the relatedness of the added species to the focal species. When there is turnover, adding species with an intermediate level of relatedness (e.g. equivalent to adding species with divergence to humans similar to mouse, rat, etc.) leads to a large increase in power per added species. There is almost no added value when adding highly diverged species (e.g., adding species with divergence to humans similar to lamprey or zebrafish). If the added species are too closely related, then power increases steadily but only very slowly (e.g., adding primate data such as baboons, macaques, or marmosets). Roughly, the increase in power when adding the mouse sequence is twice the increase in power of adding a primate sequence. Finally, we also note that adding species that are closely related to an already sampled species (e.g. adding mouse data when rat is already included) again does not substantially increase power.

These results are now summarized in a newly added supplementary text (S2 Text). Further, we refer to this tradeoff in the revised manuscript (Discussion, lines 575-580):

"...computing conservation scores from closely related species with a shallow phylogenetic relationship is advantageous since the genomes have a highly correlated functional state and are readily alignable to the focal species. However, if the overall tree size is too small, then conservation (i.e., a lack of substitutions) is harder to detect and power is low. This leads to a tradeoff between tree size and relatedness between the included species (see also S2 Text)."

########
15) p.18, “We next fit a mixture distribution to the empirical GERP score distributions…” – looking into the methods, it was not clear how the mixture model is constructed – there is no mention of algorithm, parameters, variables. It is challenging to access what the significance is here.

We thank the reviewer for pointing out the need to provide a better description of the mixture model. We now provide a more detailed and logical description of the model, including the parameters that we are inferring (the proportions of sites in the neutral, constrained, and turnover categories), and how we go about this inference in the Methods of the revised manuscript. Specifically, we write, (lines 684-721):
“*The mixture model includes three categories of mutations. The proportion of sites in each category are the parameters we estimate from the model. The first category consists of sites where mutations are neutrally evolving across the entire phylogenetic tree (category*

N). To generate the GERP scores under this model we simulated genetic data along the 36 species phylogeny using pyvolve *and* gerpcol *as described above (*Simulating deleterious substitutions along a phylogenetic tree *and* Estimating the rate of substitutions with GERP++*). The second category consists of sites where mutations are consistently under purifying selection across the entire tree, such that substitutions would not occur and GERP scores would show the maximum value (category C). The third category consists of sites that had experienced functional turnover or had changed selection coefficients over the timescale of mammalian evolution (category TO). To generate GERP scores under this category, we used the turnover model of Rands et al. as described above (*Simulations under the turnover model*), with a rate parameter of turnover that was estimated from intergenic data [25].*

Because GERP scores do not follow a common probability distribution [19], we used a nonparametric approach. We separately fit a kernel density to the empirical distribution of GERP scores and as well as to the GERP scores simulated under a particular mixture model (see below). The density was estimated in R using the density function with a bandwidth of 0.05, a number of grid points of 5000, and a gaussian kernel. After calculating a kernel density of both empirical and simulated distribution, we then assessed the fit of different models using the overlap statistic (O_{Model}) of the distribution of standardized GERP scores observed in the data with the distribution of standardized GERP scores under the model (S8 Fig). O_{Model} was measured from kernel density estimation of both distributions. The density was estimated in R using the density function with a bandwidth of 0.05, a number of grid points of 5000, and a gaussian kernel. After calculating a kernel density of both empirical and simulated distribution, O_{Model} is calculated as two minus the sum of the absolute difference in density of model versus data at each of the 5000 grid points on the standardized GERP score axis, multiplied by 0.0006, the distance between two neighbouring grid points. Thus, O_{Model} is measuring the amount to which the two probability densities overlap. A value of O_{Model} of zero indicates no overlap between the two distributions, a value of one indicates perfect overlap, a value between zero and one indicates intermediate overlap. The parameters for each mixture model (i.e., the mixing proportions of sites in category N, C, and TO) were chosen such that they maximize O_{Model}, i.e. such that the model fits optimally to the data. Maximization was achieved by an exhaustive grid search over a dense grid on the mixing proportions of the three components N, C, and TO, constrained on the proportions adding to one. The mixing proportions that lead to the largest overlap between model and data were defined as the estimates of the proportions. Simulations suggest that the estimates are accurate and unbiased (S9 and S10 Fig). Summing over the different tree sizes weighted by their relative proportions across the genome was used to estimate the genome-wide proportion of sites in each category.".

########
16) p.19, "We next assessed how well a model fits the data by examining the overlap of the distribution of standardized GERP scores observed in the data with the distribution of standardized GERP scores under a certain model" – the "certain model" here threw me

off as I am not sure what it is. Looking into methods, while there are details how model overlaps were assessed, I am not sure where the “certain model” is described.

The “certain model” referred to here is the mixture distribution. We agree that this was unclear in the previous version of the manuscript. We have replaced “certain model” to “mixture distribution”. That, combined with our improved description of the mixture model and how the inference was done (as described in our response to the point above) should alleviate confusion on this topic.

########
17) p.22, estimate of the proportion of human genome under purifying selection – consider the assumptions going into the model (still not sure how it is constructed as pointed out above), how do they impact this estimate? Why is this estimate necessarily more accurate compared to earlier ones?

In the revised manuscript we now included additional assessments of the robustness of our inference. We evaluated different models of nucleotide evolution and selection as well as variation in population size across the phylogenetic tree. These new results are now summarized in SI Text 1 (Model assumptions and robustness of inference). We also investigated how missing data affects bias and precision of estimating model parameters (S9 and S10 Fig), and how slightly deleterious mutations as estimated in Torgerson et al. (2009) would translate into GERP scores and therefore affect our inference (S4 Fig).

Our estimate of the proportion of the human genome under purifying selection should be more accurate compared to previous estimates because we include an explicit model of functional turnover. We show that including turnover results in a better fit to the data than models without turnover. We now state this explicitly in the Discussion of the revised manuscript but also point to factors that might potentially lead to underestimation of the true proportion of the human genome under purifying selection. See lines 498-519, where we write:

“*Our estimate that 4.51% of noncoding sites in the human genome experience deleterious mutations is in line with previous estimates based on conservation patterns (Ponting and Hardison 2011). However, it is most likely an underestimate of the fraction of functional sequence, for several reasons. First, our analysis does not detect functional sequences that are evolving very rapidly and/or are subjected to positive selection [49]. Positive selection increases divergence above neutral levels and thus would lead to negative GERP scores that are interpreted as neutral in our approach. Second, GERP scores are based on a neutral reference tree with branch lengths estimated from four-fold degenerate sites [19]. However, there are indications that synonymous sites of vertebrate genomes are also subject to purifying selection [50]. For example, it was shown that the overall divergence between chimpanzees and humans is 39% lower at four-fold degenerate sites than at intergenic sites [51]. Thus, the rate of evolution at four-fold degenerate sites is likely an underestimate of the rate of neutral evolution at intergenic*

sites. Using four-fold degenerate sites as neutral reference is, therefore, a conservative approach as it biases the neutral GERP score distribution to negative values and purifying selection has to be strong enough to overcome this bias. Finally, our estimate of the fraction of sites under purifying selection does not measure selection against insertions or deletions (indels). For example, indels may induce frameshifts in coding regions or secondary structure changes in RNAs, suggesting that stronger purifying selection may often act upon them than on nucleotide changes in the same region. This might explain the discrepancy between our estimates of the fraction of functional sequence and a recent estimate based on indels that suggests that about 7% of the noncoding human genome is subject to negative selection [25].".

We again apologize for the lack of clarity in the description of the mixture distribution. The revisions to the Methods in response to the previous comments should clarify what we did and consequently, the significance of our estimates.

References

Asthana, Saurabh, Mikhail Roytberg, John Stamatoyannopoulos, and Shamil Sunyaev. 2007. "Analysis of Sequence Conservation at Nucleotide Resolution." *PLoS Computational Biology*. https://doi.org/10.1371/journal.pcbi.0030254.

Bainbridge, Matthew N., Min Wang, Yuanqing Wu, Irene Newsham, Donna M. Muzny, John L. Jefferies, Thomas J. Albert, Daniel L. Burgess, and Richard A. Gibbs. 2011. "Targeted Enrichment beyond the Consensus Coding DNA Sequence Exome Reveals Exons with Higher Variant Densities." *Genome Biology* 12 (7): R68.

Boffelli, Dario, Jon McAuliffe, Dmitriy Ovcharenko, Keith D. Lewis, Ivan Ovcharenko, Lior Pachter, and Edward M. Rubin. 2003. "Phylogenetic Shadowing of Primate Sequences to Find Functional Regions of the Human Genome." *Science* 299 (5611): 1391–94.

Mouse Genome Sequencing Consortium. 2002. "Initial Sequencing and Comparative Analysis of the Mouse Genome." *Nature*. https://doi.org/10.1038/nature01262.

Cooper, Gregory M., Eric A. Stone, George Asimenos, NISC Comparative Sequencing Program, Eric D. Green, Serafim Batzoglou, and Arend Sidow. 2005. "Distribution and Intensity of Constraint in Mammalian Genomic Sequence." *Genome Research* 15 (7): 901–13.

Davydov, Eugene V., David L. Goode, Marina Sirota, Gregory M. Cooper, Arend Sidow, and Serafim Batzoglou. 2010. "Identifying a High Fraction of the Human Genome to Be under Selective Constraint Using GERP++." *PLoS Computational Biology* 6 (12): e1001025.

Graur, Dan, Yichen Zheng, Nicholas Price, Ricardo B. R. Azevedo, Rebecca A. Zufall, and Eran Elhaik. 2013. "On the Immortality of Television Sets: 'Function' in the Human Genome according to the Evolution-Free Gospel of ENCODE." *Genome Biology and Evolution* 5 (3): 578–90.

Kimura, M. 1962. "On the Probability of Fixation of Mutant Genes in a Population." *Genetics* 47 (June): 713–19.

Lanfear, Robert, Hanna Kokko, and Adam Eyre-Walker. 2014. "Population Size and the Rate of Evolution." *Trends in Ecology & Evolution* 29 (1): 33–41.

Lawrie, David S., and Dmitri A. Petrov. 2014. "Comparative Population Genomics: Power and Principles for the Inference of Functionality." *Trends in Genetics: TIG* 30 (4): 133–39.

Margulies, Elliott H., Mathieu Blanchette, NISC Comparative Sequencing Program, David Haussler, and Eric D. Green. 2003. “Identification and Characterization of Multi-Species Conserved Sequences.” *Genome Research* 13 (12): 2507–18.

Miller, Webb, Kateryna D. Makova, Anton Nekrutenko, and Ross C. Hardison. 2004. “Comparative Genomics.” *Annual Review of Genomics and Human Genetics* 5: 15–56.

Nielsen, Rasmus, and Ziheng Yang. 2003. “Estimating the Distribution of Selection Coefficients from Phylogenetic Data with Applications to Mitochondrial and Viral DNA.” *Molecular Biology and Evolution* 20 (8): 1231–39.

Pollard, Katherine S., Melissa J. Hubisz, Kate R. Rosenbloom, and Adam Siepel. 2010. “Detection of Nonneutral Substitution Rates on Mammalian Phylogenies.” *Genome Research* 20 (1): 110–21.

Romiguier, Jonathan, Vincent anwez, Emmanuel J. P. Douzery, and Nicolas Galtier. 2010. “Contrasting GC-Content Dynamics across 33 Mammalian Genomes: Relationship with Life-History Traits and Chromosome Sizes.” *Genome Research* 20 (8): 1001–9.

Rosenberg, Michael S., Sankar Subramanian, and Sudhir Kumar. 2003. “Patterns of Transitional Mutation Biases within and among Mammalian Genomes.” *Molecular Biology and Evolution* 20 (6): 988–93.

Siepel, Adam, Gill Bejerano, Jakob S. Pedersen, Angie S. Hinrichs, Minmei Hou, Kate Rosenbloom, Hiram Clawson, et al. 2005. “Evolutionarily Conserved Elements in Vertebrate, Insect, Worm, and Yeast Genomes.” *Genome Research* 15 (8): 1034–50.

Spielman, Stephanie J., and Claus O. Wilke. 2015. “Pyvolve: A Flexible Python Module for Simulating Sequences along Phylogenies.” *PloS One* 10 (9): e0139047.

Torgerson, Dara G., Adam R. Boyko, Ryan D. Hernandez, Amit Indap, Xiaolan Hu, Thomas J. White, John J. Sninsky, et al. 2009. “Evolutionary Processes Acting on Candidate Cis-Regulatory Regions in Humans Inferred from Patterns of Polymorphism and Divergence.” *PLoS Genetics* 5 (8): e1000592.

Upham, Nathan S., Jacob A. Esselstyn, and Walter Jetz. 2019. “Inferring the Mammal Tree: Species-Level Sets of Phylogenies for Questions in Ecology, Evolution, and Conservation.” *PLoS Biology* 17 (12): e3000494.

Yang, Z. 1997. “PAML: A Program Package for Phylogenetic Analysis by Maximum Likelihood.” *Computer Applications in the Biosciences: CABIOS* 13 (5): 555–56.

———. 2007. “PAML 4: Phylogenetic Analysis by Maximum Likelihood.” *Molecular Biology and Evolution*. https://doi.org/10.1093/molbev/msm088.
